# Exploring the knowledge, explanatory models of illness, and patterns of healthcare-seeking behaviour of Fang culture-bound syndromes in Equatorial Guinea

**DOI:** 10.1371/journal.pone.0201339

**Published:** 2018-09-07

**Authors:** Raquel Jimenez Fernandez, Inmaculada Corral Liria, Rocio Rodriguez Vázquez, Susana Cabrera Fernandez, Marta Elena Losa Iglesias, Ricardo Becerro de Bengoa Vallejo

**Affiliations:** 1 Facultad de Ciencias de la Salud, Universidad Rey Juan Carlos, Avda. de Atenas, Alcorcón, Madrid, Spain; 2 Escuela Universitaria Enfermería, Fisioterapia y Podología, Facultad de Medicina, Universidad Complutense de Madrid, Avenida Complutense, Madrid, Spain; Agencia de Salut Publica de Barcelona, SPAIN

## Abstract

In 1994, the Diagnostic and Statistical Manual of Mental Disorders (DSM-IV) included “culture-bound syndromes” in its classification of psychiatric disorders and associated them with disease processes that manifest in behavioural or thought disorders that develop within a given cultural context. This study examines the definitions, explanatory models, signs and symptoms, and healthcare-seeking behaviours common to Fang culture-bound syndromes (i.e., *kong*, *eluma*, *witchcraft*, *mibili*, *mikug*, and *nsamadalu*). The Fang ethnic group is the majority ethnic group in Equatorial Guinea. From September 2012 to January 2013, 45 key Fang informants were selected, including community leaders, tribal elders, healthcare workers, traditional healers, and non-Catholic pastors in 39 of 724 Fang tribal villages in 6 of 13 districts in the mainland region of Equatorial Guinea. An ethnographic approach with an emic-etic perspective was employed. Data were collected using semi-structured interviews, participant observation and a questionnaire that included DHS6 key indicators. Interviews were designed based on the Cultural Formulation form in the DSM-5 and explored the definition of Fang cultural syndromes, symptoms, cultural perceptions of cause, and current help-seeking. Participants defined “Fang culture-bound syndromes" as those diseases that cannot be cured, treated, or diagnosed by science. Such syndromes present with the same signs and symptoms as diseases identified by Western medicine. However, they arise because of the actions of enemies, because of the actions of spirits or ancestors, as punishments for disregarding the law of God, because of the violation of sexual or dietary taboos, or because of the violation of a Fang rite of passage, the *dzas*, which is celebrated at birth. Six Fang culture-bound syndromes were included in the study: 1) *Eluma*, a disease that is targeted at the victim out of envy and starts out with sharp, intense, focussed pain and aggressiveness; 2) W*itchcraft*, characterized by isolation from the outside, socially maladaptive behaviour, and the use of hallucinogenic substances; 3) *Kong*, which is common among the wealthy class and manifests as a disconnection from the environment and a lack of vital energy; 4) *Mibili*, a possession by evil spirits that manifests through visual and auditory hallucinations; 5) *Mikug*, which appears after a person has had contact with human bones in a ritual; and 6) N*samadalu*, which emerges after a traumatic process caused by violating traditions through having sexual relations with one’s sister or brother. The therapeutic resources of choice for addressing Fang culture-bound syndromes were traditional Fang medicine and the religious practices of the Bethany and Pentecostal churches, among others. Among African ethnic groups, symbolism, the weight of tradition, and the principle of chance in health and disease are underlying factors in the presentation of certain diseases, which in ethno-psychiatry are now referred to as culture-bound syndromes. In this study, traditional healers, elders, healthcare professionals, religious figures, and leaders of the Fang community in Equatorial Guinea referred to six such cultural syndromes: *eluma*, *witchcraft*, *kong*, *mibili*, *mikug*, and *nsamadalu*. In the absence of a multidisciplinary approach to mental illness in the country, the Fang ethnic group seeks healthcare for culture-bound syndromes from traditional healing and religious rites in the Evangelical faiths.

## Introduction

Coupled with rising immigration, the growth of cultural diversity and globalization led to the inclusion of “culture-bound syndromes” in the DSM-IV, first published in 1994. This category was more recently relabelled "cultural syndromes" in the DSM-V [1, 2] for psychiatric disorders [3, 4]. Mental health professionals in Western countries often identify nonspecific symptoms, "patterns of aberrant behaviour," health problems, and altered thinking, which patients attribute to a symbolic scheme, a social representation of disease, or cultural patterns rather than to a biomedical construct of disease. In none of these cases are such schemes related to the diagnostic categories published in the DSM-V[5–7]. There have been numerous examples of such cultural syndromes in Latino cultures: *mal de ojo* (evil eye), *susto* (fright), or *nervios* (nerves). For other cultures, we might mention *koro* in eastern Asia [8], *latah* in Malaysia, or *zar*spirit [9–11] which are similar to the syndromes presented here.

In his treatise "The Anthropology of Disease," François Laplantine [12] wrote, *“in all societies*, *along with implicit concepts about disease*, *we find interpretive models constructed by different cultures*.*"* He argues that we have successively moved from theological and legal explanations to medical and biological ones and ultimately to a multidimensional aetiology in which the social sphere predominates. Each social group in each generation concentrates its efforts on "the cause par excellence of disease," an understanding that is continuously forged from observation, life lessons, trial and error, and success and failure in contact with healthcare systems [12]. Each culture defines what “health” is for its members and determines the aetiology of disease. Each affliction is defined and located, and the appropriate remedies to address the problem, both medically and socially, are determined [13]

There are observable commonalities among sub-Saharan African cultures; among them is the aetiological model of disease. “Getting sick" is a warning of having committed an offense or violation and requires redress of community norms [12]. In addition, for sub-Saharan African peoples, knowledge has its origin in the ancestors and is transmitted by the elders to the younger generations, which means that there are few opportunities for it to undergo transformation. Any attempt to question these arguments is penalized by taboos, rites, and prohibitions [14].

The Fang are an African ethnic group of Bantu origin who initially occupied the Sudanese plateau of Bahr-el-Gazal[15][16]. They currently are found in Brazzaville (Congo), in central and southwest Cameroon, in Gabon, on Príncipe Island, and in Equatorial Guinea [15]. The social structure of the Fang ethnic group is defined traditionally as conservative, hierarchical, and patriarchal [17]. Main decisions, policies, religion, rites, traditions and ceremonial initiation rites are handled by the Fang family, particularly elders and men, along these lines [15].

The family structure of the Fang group is arranged into clans. The names of these clans have a nexus with animals, occupations, or mythology. Every clan forms an extended family that shares ties of blood and geographical location [18]. Polygamy is allowed and is practised to an extent. A woman must leave her Fang village when she marries and live beside her husband’s family. Their children are named after the father’s clan. If she ever wants to divorce from her husband, she must return her dowry to the husband’s family [15]. Every Fang clan has its taboos, legends, and a traditional leader. The leader must take responsibility for justice, playing the role of umpire or judge in resolving conflicts concerning matters such as land ownership, arranging marriage ties with other clans, and choosing the therapeutic course in the event of disease. He can identify signs of folk illness and send patients to the traditional healer for treatment, or he might decide they should go to the hospital [19].

The Fang are Animists by definition; animals, plants, all elements of nature, and inanimate objects are a projection of the spirits of their ancestors [20]. Ancestors play an important role in Fang Animistic beliefs; ancestors are asked, for example, for help, favours, protection, and advice, and the Fang believe that their spirits remain alive [21]. In closed circles, Fang people continue practising witchcraft through the religious sects of *mbueti* and *ngui*.

For the Fang people, health, illness, and death are states that appear to be consequences of the intervention of a "force" directed by some malignant entity that serves to provide benefits to a third person [21].However, the cause occasionally tends to stray from the scientific principle of cause and effect and comes very close to Jung’s principle of synchronicity – any type of events without conclusive cause (nature events: floods, burnings… burglary, arguments between relatives or neighbours, loss of employment…) happen at the same time as a disease or a death [22–24].

The aim of this paper is to review common Fang cultural syndromes in Equatorial Guinea and describe the Fang syndromes, symptom presentation and healthcare-seeking behaviour from diverse perspectives: Fang community leaders, Fang tribal elders, healthcare workers, Fang traditional healers and non-Catholic pastors.

We must understand the cultural context of disease to avoid erring in diagnosis or treatment, to improve quality of life, and to facilitate the integration of patients affected by Fang cultural syndromes. Healthcare professionals must explore and share knowledge about the nomenclature, aetiological models, and signs and symptoms of these types of cultural syndromes to develop effective tools to address them. It might even be possible to discover elements in the environment of the disease that provide tools for progress, drawn from fields other than healthcare. This little-explored topic provides relevant information on different culture-bound syndromes in the Fang group. A review of the literature indicates that this study is the first to report on culture-bound syndromes in the Fang ethnic group.

## Materials and methods

### Study area and population

Equatorial Guinea is located in Western Central Africa, with a surface area of 25,667 sq. km. The area is divided into a mainland region and an insular one and supports a population of 759,451 people. The population is mostly Catholic due to 190 years of Spanish rule, although there are other Protestant faiths, and in recent years, Protestant groups have steadily increased. Fang (85.7%), Bubi (6.5%), and Ndowe (3.6%) are the largest ethnic groups. Approximately 59,63% are under the age of 25, and only 39.9% of the total population lives in an urban area; the majority of Fang inhabitants in the mainland region work on subsistence farms in the tribal villages without clean sources of drinking water [25].

Equatorial Guinea’s economy has grown considerably due to large offshore oil and gas reserves. Equatorial Guinea is the richest country in Africa [25], [26–27]. In the last ten years, achievement of improvements in social indicators (health expenditure, poverty rate, youth education…) has occurred due to the modernization of public healthcare facilities and education infrastructure, the expansion of immunization coverage, and the implementation of 2 hydroelectric plants.[27]. However, there has also been substantial improvement in spending on public education and healthcare (3,2%, 5% of the GNI in 2014) [28]. Conversely, only one of the eight Millennium Development Goals could be achieved by 2015 – universal primary education with a 76% net primary enrolment rate. Although the literacy rate increased to 97% in 2011, the secondary school enrolment rate is 31%, and the higher education enrolment rate is less than 10% [27]

The UNDP ranked Equatorial Guinea 138th in the Human Development Index 2014. The GNI per capita was approximately $18.635 26], which might be the largest gap between GNI and the Human Development Index in the world. There is an infant mortality rate of 80 deaths per 1,000 live births [28] and a maternal mortality ratio of 240 deaths per 100,000 live births [26, 29–31]. Today four models have been established in the Equatorial Guinean healthcare system: 1) private healthcare system: High quality in health coverage, expatriated doctors and modern hospitals; 2) a social and community health insurance for Guinean employees, called “INSESO”; 3) a public healthcare system, the government supports professionals and the maintenance of hospitals and materials, treatment and diagnosis are spending by out-of pocket payment of patients and relatives and; 4) programmes financed through charity foundations, religious organizations.

The public health facilities in the mainland region comprise health posts, health centres, 13 hospital districts and one regional hospital located in the city of Bata. In 2016, only 2 Cuban psychiatrists served the entire population in the Regional Hospital of Bata, and there are 2 mental health religious nurses in Angokon Benito Menni Mental Health Center [32–33]. There are also private health clinics, Chinese medicine clinics, traditional medicine posts, drug stores and modern pharmacies without regulations. Traditional medicine is an valued resource among the Equatorial Guinea population, particularly among the Fang population [32]

### Participants

Forty-five Fang key informants belonging to 22 Fang clans *(Data in Table 1)* were selected purposively, (*Data in Table 2)* including 6 community leaders, 19 tribal elders, and 20 agents who were associated with the healthcare system in Equatorial Guinea (8 healthcare workers, 10 traditional healers, and 2 Protestant pastors). Participants gave their informed consent and had the right not to participate.

**Table 1:** Fang clans and category of participants (n = 45).

FANG CLANS	CODE	CATEGORY
**ESAWONG CLAN**	E2	Traditional healer
	E3	Pastor
	E13	Healthcare worker
	E14	Fang Community leader
**OSERENGON CLAN**	E7	Elder
	E19	Elder
	E24	Healthcare worker
	E46	Elder
**ESANVUS CLAN**	E4	Pastor
	E18	Elder
**ONVANG CLAN**	E8	Traditional healer
	E40	Fang Community leader
	E45	Healthcare worker
**ANVOM CLAN**	E28	Elder
	E10	Fang Community leader
	E9	Healthcare worker
	E26	Fang Community leader
	E58	Elder
	E34	Traditional healer
**YESUK CLAN**	E17	Elder
	E27	Elder
**EFAK CLAN**	E22	Elder
**YEMBAN CLAN**	E38	Elder
**YEMANDJIM CLAN**	E39	Elder
**YENKEN CLAN**	E56	Elder
**NGAMA CLAN**	E57	Fang Community leader
**ESUMU CLAN**	E32	Elder
**MBON CLAN**	E23	Elder
**ESISIS CLAN**	E51	Traditional healer
**ESAKORA CLAN**	E55	Elder
**NSOMO CLAN**	E25	Elder
	E44	Healthcare worker
**ANGOK CLAN**	E33	Elder
**NCODJEN CLAN**	E16	Healthcare worker
	E20	Elder
**NDONG CLAN**	E41	Traditional healer
	E42	Traditional healer
**OYAK CLAN**	E48	Fang Community leader
	E49	Elder
**ESAVENG CLAN**	E35	Traditional healer
**OBUK CLAN**	E43	Healthcare worker
	E47	Healthcare worker
	E50	Traditional healer
	E52	Traditional healer
	E53	Traditional healer

**Table 2:** Categories of participants (n = 45).

CATEGORY	*N*	%
Elder	19	42.2
Fang Community leader	6	13.3
Healthcare worker	8	17.9
Traditional healer	10	22.2
Pastor	2	4.4
**Total**	**45**	**100**

### Participant recruitment

Sampling was conducted using snowball sampling techniques and done in a purposeful manner. Recruitment occurred in two phases. First, the research team selected clans with many dependents living in the area. The external facilitator in each district and the village chairperson recommended representative tribal elders. The head of traditional healers in Equatorial Guinea recommended the most representative Fang traditional healers or pastors, and the chief medical officer of the hospitals recommended the healthcare workers. The following general criteria were applied to the candidates in the second phase of recruitment: being of Fang ethnicity, being of Equatorial Guinea nationality, having an address in the mainland region, accessibility, participant's willingness to participate in open discussions, and being a leader for the Fang community in this area.

### Data collection

This study was conducted in September 2012 to January 2013, in 21 of 724 Fang tribal villages, in 6 of 13 districts: Ebebiyin, Bata, Mongomo, Akonibe, Niefang and Kogo, in the mainland region of Equatorial Guinea, in which “Fang” is the most important ethnic group. All data were collected by a team of 4 people – 1 foreign and 3 local. Prior to the investigation, the entire team had been trained in qualitative techniques and in the vernacular language. Each member of the team changed roles when they began in a new district: interviewing, note-taking or participating observation.

Prior to data collection, participant observation was done by the study team. The team lived in the mainland region for 3 months, immersing themselves in the Fang cultural context and describing daily routine activities of the Fang participants’ way of life: foods, hygiene, education, entertainment, healthcare seeking, oral expressions, rules, and traditions such as burying, wedding, engagement, childbirth, and relationships among all members of the Fang community. The research team first sought permission to conduct the study from the regional authorities. Furthermore, they selected a Fang representative in each district, called the “external facilitator.” The external facilitators are people respected by the community through their professional careers in healthcare or education programmes. We were allowed access to the community, and we mobilized the key informants for the interviews as recommended by Atim et al. [34–36].

This study used an ethnographic approach with an emic-etic perspective, using the conceptual model of Harris [37–39]. This strategy involved living with Fang families in the villages for weeks (similarly to the methods of Akinlua et al. [40]. The emic-etic perspective allowed us to discuss different kinds of knowledge and complementary data central to our investigation – for example, healthcare-seeking behaviours, understanding the sense of life from a Fang perspective following Simons D et al. [41] through an objective, outsider perspective (etic) following Ruanoa. L. et al. [42] and Leseth A.B [43], and from the participants’ point of view (emic). Data collection was conducted using two qualitative methods: semi-structured interviews and participant observation.

Semi-structured interviews were usually conducted in the language of the participant’s choice, Spanish or Fang as Bennett et al. [44], by a member of the research team, with the assistance of an interpreter if necessary. Recorded and filmed interviews lasted from 15–45 minutes. The 5 initial interviews were piloted and reviewed by members of the research team to ensure high quality and consistency as recommended by Jones et al. [45]. When exploring other population groups (i.e., young people and women), the Fang women who were consulted showed little interest in being involved in the project. Interviews were designed using the “Cultural Formulation” form in DSM-5, as recommended by Jacob K.S et al. [46] and explored three categories: 1) knowledge about the cultural definition of the problem: definition, symptoms, and course; 2) cultural perceptions of cause, context, and support; and 3) current help-seeking behaviours. One member of the research team did the participant observation during the interview. The participant observer was an expert on the Fang way of speech, mannerisms, and Fang onomatopoeias. Data gathered from the observations were written in a field diary. Qualitative strategies were accompanied by quantitative methodology. We use some key indicators (101–127) of the *Demographic and health surveys for the United States Agency of Internacional Development*, *Agency 2008*–*2013* (DHS6)questionnaire [47], which was designed to provide a demographic profile of the participants – for example, housing conditions, shelter materials, electricity, and access to safe water sources.

### Data analysis

Twenty-two of the 45 interviews were translated from Fang to Spanish. The translations were checked 3 times – by the Fang research team, by participants and by another Fang person not involved in performing the research [48].Two authors (RJ and ML) independently identified concepts that can constitute codes [49, 50] from relevant literature, transcripts of interviews and the field diary notes written by the participant observer. Similar categories and themes were identified by 2 members of the research team, and according to the research objectives, an inductive process was used until saturation was achieved [51]. Data saturation was discussed by the research team. We then developed analytical codes within the following broad categories: definition, signs and symptoms, explanatory model and cultural context of Fang culturally bound syndromes, and healthcare-seeking behaviour. The data were summarized systematically according to how likely they are to occur or by the identification of any discrepant or similar answers. Thematic content emerging from interviews was triangulated in several ways: 1) across respondents of different ages, districts, jobs, and religions; 2) across the research team members and other researchers who worked on the project; and 3) between interviewers and participant observation. Findings were also fed back and validated in the country by participants [44, 52, 53]. At the same time, data were imported and analysed using NVivo 9 Qualitative Data Analysis software (QSR International Pty Ltd. Cardigan UK) [48]. The study adhered to COREQ guidelines [54] and the Lincoln and Guba’s alternative criteria: credibility, dependability, confirmability, and transferability [55, 56].

### Ethical considerations

The study was approved by the Directorate General of Pharmacy and Traditional Medicine of Ministry of Health and Social Well-being of Equatorial Guinea and Ethic and Regulatory Issues, Clinic Research Projects Committee, La Paz, Medical Centre in Bata, composed of data managers and chief doctors from Israel.

Participants were informed of the aim of the study, the types of question to be asked, and how results would be distributed through publication in scientific journals and mainstream media. The research team worked to ensure that the Fang cultural legacy and identity were protected it. Anonymity and confidentiality were guaranteed, and participants had the right to refuse or stop the interview at any time. Informants’ data were coded and not publicly available. Verbal rather than written consent was preferred by most participants; therefore, the interviewer read the informed consent document to the participant in a familiar place surrounded by relatives. Elders and Fang traditional healers tend to be illiterate or have visual or hearing problems [48]. Interviews were conducted at a place of the participants’ choice, at home or outdoor in the tribal village. Upon completion of the project, external facilitators, pastors, Fang community leaders, Fang traditional healers and healthcare workers were provided monetary compensation for involvement in the study. Elders received a range of products from food to hygienic products.

## Results

Of the 45 participants selected for the interviews, 7 were women *(Data in Table 3)*. The ages of the participants ranged from 26 years to 95 years. Twenty-seven respondents lived in Fang tribal villages and 18 in urban areas (in order from larger to smaller populations): 7 from the region capital (Bata), 5 from the provincial capitals (Mongomo, Ebebiyin), 6 from the district capitals (Kogo, Niefang, Akonibe). Table 4lists the basic household data of participants. Forty-four per cent live in wooden houses, 75.6% have electricity in their homes, and 31.1% own vehicles.

**Table 3:** Socio-demographic profile of participants (n = 45).

CONCEPT	*N*	%
**GENDER**		
Female	7	15.6
Male	38	84.4
Total	45	100
**AGE**		
25–40	2	4.4
41–50	4	8.9
51–60	6	13.,3
61–70	16	35.7
71–80	11	24.4
81–90	4	8.9
91–100	2	4.4
Total	45	100
**MARITAL STATUS**		
Married	13	28,9
Married polygamous	26	57,8
Widows	4	8,9
Singles	2	4,4
Total	45	100
**RELIGION**		
Catholic	39	86,8
Evangelic confession	2	4,4
Bethany confession	3	6,6
Others	1	2,2
Total	45	100

**Table 4:** Household data of participants (*n* = 45).

CONCEPT		*n*	%
**RESIDENCE**			
Capital Region		7	15,6
Capital Province		5	11,1
Capital District		6	13,3
Fang tribal village		27	60
Total		45	100
**DISTRICT**			
Mongomo		10	22.2
Kogo		11	24,4
Akonibe		7	15,6
Niefang		8	17.8
Bata		9	20
Total		45	100
**MAIN MATERIAL OF THE EXTERIOR WALL**			
Covered adobe		0	0
Wood planks		20	44.4
Cement blocks		25	55.6
Total		45	100
**HOUSEHOLD FACILITY**			
Electricity	Yes	34	75.6
	No	11	24,4
		
TV	Yes	34	75.6
	No	11	24,4
		
Refrigerator	Yes	35	77.8
	No	10	22.2
		
Radio	Yes	44	97.8
	No	1	2,2
		
Vehicle	Yes	14	31.1
	No	31	68.9
**SOURCE OF HOUSEHOLD DRINKING WATER**			
Public tap	Yes	9	20
	No	36	80
		
Dug well protected	Yes	12	26.7
	No	33	73.3
		
Open cistern	Yes	10	22.2
	No	35	77.8
		
Surface water / river	Yes	14	31.1
	No	31	68.9
**MAKING THE WATER SAFE TO DRINK**			
Nothing	Yes	23	51.1
	No	22	48.9
**TYPE OF TOILET FACILITY**			
Piped sewer system	Yes	0	0
	No	45	100
		
Septic tank	Yes	16	35.5
	No	29	64.5
		
Pit latrine with slab	Yes	16	35.5
	No	29	64.5
		
Open pit latrine	Yes	9	20
	No	36	80

Finally, the research team structured the qualitative findings to address the following categories: 1) definition, 2) sign and symptoms, 3) explanatory model and cultural context of Fang culture-bound syndromes and 4) healthcare-seeking behaviours. Below are examples of narratives that best represent each category.

### Definition

Study participants tended to conceptualize Fang culture-bound syndromes by considering the effectiveness of the procedures that Western medicine uses to correct them. For example,

*“Those diseases that cannot be cured, treated, or diagnosed by science …*” *E4 Esanvus clan, pastor*

"*When the scientific medical treatment is administered*, *it doesn’t cure them…*" *E47 Obuk clan healthcare worker*

Participant observers also revealed that “culturally bound syndromes” are popularly defined as a set of signs and symptoms that do not respond favourably to Western medical treatment …, even when this lack of results occurs because the necessary human, material, or diagnostic resources are not available.

Traditional healers, elders commented that Fang cultural syndromes are also reported in other non-Fang countries and even in Western countries:

*Fang folk illnesses such as witchcraft*,eluma, or mikug “*will be given other names*, *but they can also be found in other countries*, *such as Cameroon*, *France*, *or Spain” E51 Esisis clan*, *traditional healer*

*“He’s been for 4 months in Cameroon, visiting some different places. He’s seen the same folk illnesses …*” *E48 Efak clan, Fang community leader*

*“My brothers used to go from Gabon to Cameroon, and those of them who are working at the bigger companies, they’ve been seeing on the white population …*” *E7. Oserengon clan, elder*

### Signs and symptoms of Fang culturally bound syndromes

In their discourse Fang community leaders, elders, traditional healers, and healthcare professionals assembled an amalgam of signs and symptoms that outline these six Fang cultural syndromes: *eluma*, *witchcraft*, *kong*, *mibili*, *mikug*, and *nsamadalu*. In general, they consider the signs and symptoms that patients present in these processes to be similar to those of the diseases of Western medicine.

"*The Fang folk illnesses present the same signs and symptoms that may be manifested in the diseases of modern medicine*." *E48 Oyak clan*, *Fang community leader*

"*A person who has hallucinations, for us, bad dreams – it means a folk illness.*" *E44 Nsomo clan, healthcare worker*

#### Eluma

The study participants defined eluma as a "projectile" disease. It is characterized by aggressiveness, maladaptive behaviour, headaches, and acute, intense, focussed pain.

"*I had a prickling sensation in my head – very strong pains*. *I screamed at people*, *I threw things at them*, *I bit them*.*" E16 Ncodjen clan healthcare worker*

“*Pains in your back, like stinging, as if someone was sticking knives into you*." *E3 Esawong clan pastor*

“*There are usually acute pains in your side, and then screaming, running, days and days without sleep*." *E25 Nsomo clan, elder*

#### Witchcraft

This Fang cultural syndrome was deeply rooted within the cultural context of the Fang culture. It is considered the true manifestation of a Fang folk illness and is related to mental illness.

“*Witchcraft is the real Fang folk disease…" E34 Anvom clan*, *traditional healer*

Furthermore, it tends to modify one’s social behaviour.

"*You can be [left*?*] poor*, *with no wife*, *no children*, *no money*, *nothing*.*" E33 Angok clan*, *elder*

"*You don’t know where you are*, *you don’t know your family*, *you go around with no clothes on*.*" E34 Anvom clan*, *traditional healer*

"*You do things outside of the norm, you suddenly take off running, [and] you go around on the street with no clothes on, without brushing your hair, without bathing.*" *E10 Anvom clan, Fang community leader*

“*Then, your body is left limp, you don’t know anything, you can’t read, you can’t speak … you say words without joining them together [into sentences?].*" *E10 Anvom clan, Fang community leader*

"*They stop doing their tasks, they don’t take care of their children, they talk about strange things, they go out at night, [and] they don’t have any direction. Their eyes get big, but they can’t see.*” *E2 Esawong clan, traditional healer*

“*Their eyes are red; they bulge out of their sockets*." *E16 Ncodjen clan, healthcare worker*

The participant observer noted that people diagnosed with witchcraft were dishevelled and disconnected from their environment, and some of them retained a certain dilation or construction of the pupils, had dry mouth, verbalized visual hallucinations, and likely were under the effects of certain hallucinogenic substances. In Equatorial Guinea, there are geographical areas where the local people are knowledgeable about poisons derived from plants or animals. Furthermore, the anguish they suffered when practising rituals that were socially forbidden or religiously punished was obvious to the researcher conducting the observation.

#### Kong

This folk illness manifests through aberrant behaviour, lethargy, and disorientation.

” *You become half crazy, you go out to the forest at night, you eat whatever kind of meat you might find*." *E38. Yemban clan, elder*

"*You appear slow, disoriented, disconnected.*" *E3 Esawong clan, pastor.*

“*The Fang people call kong an "imported" disease*.” *E9 Anvom clan healthcare worker*.

“*Imported from the magic rites of others countries such as Cameroon …” E52*, *Obuk clan*, *traditional healer*

*“It comes from Cameroon; it’s not a “typical” Fang disease that is original to Equatorial Guinea*." *E40 Onvang clan Fang community leader*

For the participant observer, patients who had seizures or described hallucinations, disorientation, altered mobility or language have typically been diagnosed with “kong” in traditional medicine. These patients often eventually visited the Western medical practitioners, who detected dementia, late-stage syphilis or very advanced stage AIDS, with pathologies associated with blindness, encephalitis, or meningitis. Those most affected are the wealthier Fang social classes.

#### Mibili

Traditional healers and healthcare professionals describe the clinical manifestations as visual and auditory hallucinations, nocturnal nightmares, and the appearance of funeral rites in one’s thoughts:

*“When he feels that he is sitting like this*, *he hears noises on both the sides; when he walks*, *it seems that someone is following him; when he sleeps he has bad dreams*, *he sees things from the tombs*, *he believes that what he feel [is] a folk illness; ‘I have* mibili.*’” E16 Ncodjen clan*, *healthcare worker*

#### Mikug

Mikug is a culture-bound syndrome that for example progresses to changes in movement of the limbs, freezing, and a sense of disconnection from the environment:

“If *a woman was touching the idols*, *the skulls*…*she would get very sick; her body would be paralyzed and she’d be unable to move her legs or arms*.*" E55 Esakora clan*, *elder*

*“They leave you frozen; they hypnotize you. Sometimes it attacks the person’s body and weakens them; they’re lying in bed without talking, [without?] wanting to eat, looking up at the ceiling – they’re like that for days and days*.“ *E13 Esawong clan, healthcare worker*

#### Nsamadalu

Manifests through weight loss, isolation from the outside, and difficulty in vocalization. This disorder can be repeated across subsequent generations of the family.

*“They won’t go to celebrations, they can’t work, they don’t speak, and sometimes they stay in bed, without eating*.” *E32 Esumu clan, elder*

*"They are isolated from people, they’re weak, they don’t talk, they’re crying, sometimes they shout at the elders*, *and their children are born with serious health problems." E39 Yemandjim clan, elder*

*“…as a consequence, they would get skin problems or be left mute as punishment.*" *E25 Nsomo clan, elder*

### Explanatory models of Fang culture-bound syndromes

Elders, traditional healers, and healthcare workers consider these types of ailments the result of the action of forces coming from other people with whom the victim did not maintain a good relationship. For example, eluma is caused by someone who feels envious of another and thus projects a change in health onto the victim.

*“…If I am your enemy and I go to a person who has* eluma, *I can send you an illness through spiritual contact*.*" E3 Esawong clan*, *pastor*

*“…If someone is envious of another person*, *he can give you* eluma" *E35 Esaveng clan*, *traditional healer*

*“Through supernatural forces aimed at empowering them*…*” E47, Obuk clan, healthcare worker*

“*These people who don’t like you*, *they call themselves magicians*, *[and] they can prepare something for you like this*." *E50 Obuk clan*, *Fang traditional healer*

Furthermore, they might be people who are part of the family environment:

*“A relative who wishes evil on you and has given you an illness*.*"E9 Anvom clan, healthcare worker*

Fang culturally bound syndromes can also be triggered by the action of spirits or the ancestors.

#### Kong

Your spirit is being manipulated by a stronger spirit.

*“…You can draw the spirit out of his body off until death comes to him and when the body is dead*, *his spirit is gone another place*, *working bad things for the person…*” *E3 Esawong clan*, *pastor*

#### Mibili

Healthcare workers and traditional healer have described mibili as a folk illness originating from possession by evil spirits or ancestors; you and the ancestor have a rivalry going on.

“*A person possessed by evil spirits suffers from mibili*" *E35 Esaveng clan, traditional healer*

“*…Sometimes evil spirits bug us… so*, *we call it mibili…" E9 Anvom clan healthcare worker*

“*We are spiritualists; you can say that such-and-such spirit can cause disease*" *E41 Ndong clan, traditional healer*

“*An ancestor has entered their body, that person changes their voice, and they start to talk differently, or a grandfather who died in the family is inside their body – for example, a man who starts to talk in the voice of a girl*." *E45 Onvang clan healthcare worker*

"*Some ancestors can cause illnesses*." *E9 Anvom clan healthcare worker*

“*The ancestors can communicate with the alive people, and if you don´t have a good relation with them they can give you a disease*." *E49 Oyak clan elder*

#### Punishment because of flouting the law of God

The participants indicate that you can suffer culture-bound syndromes when you flout the law of God.

“*When someone has disobeyed, when they have disrespected an elder from the village, an ancestor or perhaps a relic – that can also have consequences – a serious mental illness*."*E3 Esawong clan, pastor*

*A long time ago, the meaning of folk illness was sin…*” *E56 Yenken clan, elder*

“*…The origin of folk illness is flouting, is the sin…*” *E46. Oserengon clan, elder*

The participants indicate that **y**ou are going to suffer from kong if you try to be greedy:

"*It is acquired due to ambition for money …*” *E40 Onvang clan Fang community leader*.

“*It’s attributed more often to the wealthy class …*" *E45 Onvang clan healthcare worker*

"*If you want to be a richer man, you have to do medicine…*” *E38. Yemban clan, elder*

Healthcare workers attribute this type of condition to contact with human bones.

"*Mikug* … When you c*ontact your clothes*, *your hair*, *a photo – and bones or human skulls" E13 Esawong clan*, *healthcare worker*

Participant observer has linked this expression with an ancient rite that the Fang ethnic group celebrated a long time ago, in which the bones or skull of a good person from the family, *melán*, were venerated, and family members would implore them to grant favours, to provide good crops, or to eliminate illnesses. With the arrival of the Spanish colonizers, this pagan rite was banned and became sacrilegious.

Fundamentally, folk illnesses are attributed to violations of taboos:

“*It’s a bad omen; it brings you misfortune, bad luck*." *E10 Anvom clan, Fang community leader*

“*Some person who has made a mistake involving a traditional rule, that’s the person who gets sick*." *E44 Nsomo clan, healthcare worker*

Food taboos are related to the prohibition of eating certain animals, such as “c*hid tum*." Members of the Anvom clan do not eat elephant meat, because the elephant means wealth. Members of the Ncodjen, Onvang, Esanvus, Anvom, Yemandjim, Ngama, and Yenken clans do not eat gorilla or chimpanzee meat. Members of the Esanvus clans do not eat *ñok –* a bird that sings in the forest when it hears noise. These clans report similar reasons for not eating these meats; the animals provided important information in the past, warned members of the clan of future danger when fighting with other clans, or protect them from infectious diseases.

“*If my father tells me not to eat chimpanzee, and later I do it, I may get a folk illness afterwards*." *E8 Onvang clan, Fang traditional healer*

“*They’re really not used to eating chimpanzee. In the past, when they´re going to wage war against another clan, the gorillas advised us in the forest, take care, another clan is out there, you have to back off …*” *E8 Onvang clan, Fang traditional healer*

“*The Yemandjim clan can never eat gorilla … In the past, gorilla helped them during the war*. *If they found it, they´d know that the enemies are behind it …” E39 Yemandjim clan, elder*

“*Our clan never eat gorilla*. *Gorillas used to scream when someone is nearest to die. This is a warning signal …” E57. Ngama clan, Fang community leader*

“*We don’t eat ñok meat*. *It´s an animal that signs meanwhile is listening noises…” E4, Esanvus clan, pastor*

“*Women didn’t eat fish or animals that lived in the mud due to the risk of having a difficult delivery or a child with a deformity, a child who is crazy and screams and cries because of evil spirits*." *E13 Esawong clan, healthcare worker*

Some of these prohibitions can also be specified for each individual by family elders, in conjunction with a traditional healer, in a Fang rite of passage known as “*dzas”*,which occurs a few days after birth. Healthcare professionals, elders, Fang community leaders, and traditional healers describe this ritual in uniform terms, with no difference in the details, even among Fang who belong to different clans.

“*When you’re born, there are traditional family preparations; they put prohibitions on things you should not practice throughout your entire life. If any family member violates one of these rules, a sudden illness or death may occur in the family*.” *E25 Nsomo clan, elder; E42 Ndong clan, traditional healer; E48 Oyak clan, Fang community leader; E40, Onvang clan, Fang community leader*

*"They went to the forest to gather herbs*, *they put them in a basin or a bucket*, *they add water*, *and they bathe the baby*. *They call it ‘dzas*’, *and that’s where they mark you with your prohibitions*, *the things you should stay away from during your life; and if you do*, *you run the risk of going crazy*.*” E58 Anvom clan*, *elder*

“*They prepared a basin with traditional herbs, and that’s where they put the children, and they gave them their prohibitions. Each of these herbs had some meaning. If you ever make a mistake, then the craziness would start*.” *E16 Ncodjen clan, healthcare worker*

All participants described the existence of numerous prohibitions in the clan to which they belong:

Sexual taboos: Nsamadalu: This suffering occurs after an endogamy event among members of the same Fang clan and is considered a breach of traditional rules; violators live with feelings of guilt.

“*When a brother and sister have sexual relations, or two people from the same clan …*" *E49 Oyak clan elder*

“*Fornicating with a relative brings this disease …*" *E46 Oserengon clan elder*

*“A daughter of the Esumu clan and my son here*, *from the Esumu clan – they can’t marry*. *If they marry*, *the bride and groom – they’ll start to have illnesses*, *‘nsamadalu’*.” *E32 Esumu clan*, *elder*

“*This was considered a violation of traditional rules …*” *E25 Nsomo clan, elder*

#### Witchcraft

The Fang cosmogony incorporates the concept of *evú* into the witchcraft folk illness; evú is the second soul, the evil spirit. The *evú* is activated by some elder witches or sorcerers in a witchcraft ritual called “avalaga” in childhood.

"…*Witchcraft, I don’t know if it’s spiritually when you’re born, you’re given some treatments, so you can manipulate it*." *E28 Anvom clan, elder*

*“It’s the spell that someone puts on your body*. *It's like a* “*chif"*. *It's like a second soul; evú is the battery of the witchcraft*.*" E47 Obuk clan healthcare worker*

“*Evú only feeds on blood …*” *E16 Ncodjen clan, healthcare worker*

“*… that has the power to manipulate the mind of the person, to cancel it out…*" *E33 Angok clan, elder*

“*They are indoctrinated for ‘the bad life’; avalaga is a ceremony that the wizards do, all alone*.” *E47 Obuk clan healthcare worker; E34 Anvom clan, traditional healer*

The child becomes enchanted and can practice witchcraft as an adult.

“*… so that in the future they can develop the art of witchcraft*." *E47 Obuk clan healthcare worker*

You can suffer witchcraft if you are injured and fall down during sorcery rituals; your soul will fight with another soul.

“*…If there were any disturb between the participants in a sorcery ritual, and some of them is injured*. *The wound is on the spell …”E10, Anvom clan, Fang community leader*

### Healthcare-seeking behaviours: Fang culture-bound syndromes

Participants explained that to undergo healing for culture-bound syndromes – witchcraft, non biomedical disease aetiology, or folk illness caused by breaking traditional prohibitions – people must go to a Fang traditional healer:

“*Clinical manifestations that the patients do not associate with biomedical disease aetiology are focussing on traditional healing*”.*E44 Nsomo clan, healthcare worker*

*When the clinical manifestations are not continued, it´s supposed to exist, but disappears sometimes, here´s even a hidden part of illness, the tradition is the only way to solve it…*”.*E3, Esawong clan, pastor*

"*… caused by people, with the witchcraft*" *E4 Esanvus clan, pastor*

“*It´s already a matter of witchcraft … they have to go to the typical Fang healers*." *E14 Esawong clan*, *Community leader; E49 Oyak*, *clan elder; E34 Anvom clan*, *traditional healer*

“*…sorcery is healed by Fang traditional healing …*” *E57 Ngama clan, Fang community leader*

This statement also applies to illnesses that are caused by breaking a traditional law:

“*Someone has done something they shouldn’t have done …*” *E40 Onvang clan, Fang community leader*

"*… diseases derived from the confrontation between the ancestors and the sick person*" *E52 Obuk clan, Fang traditional healer*

Finally, when a Fang culture-bound syndrome is linked to a spiritual illness, Fang people also visit Pentecostal and Bethany churches to cure it. Pastors, elders, and healthcare workers said that praying to repair the spiritual component is the common procedure to treat it.

“*Fang man is dual, he believes in the body and the soul. Spirits exist; that is the reason … he’s looking for the churches…*” *E17 Yesuk clan, elder*

“*The work of the church does not address the physical part but rather the spiritual part …*" *E3 Esawong clan, pastor*

“*They cure the evil spirits*." *E9 Anvom clan healthcare worker*

“*We’re treating evil spirits, mibili, we ask for God that the spirits can flee from the body …*” *E4 Esanvus clan, pastor*

"*The Pentecostal priests cure the madness*." *E33 Angok clan, elder*

*The Bethany faiths were praying for people who feel weakness of the flesh to cater for the influx of devil spirits, after praying for them, they recover the energy*.*E8 Onvang clan, traditional healer*

"*What they did was pray for me, [have me] confess everything I had done*." *E16 Ncodjen clan, healthcare worker*

“*If they take in a sick person, they take him, do their prayers for him, and save him*." *E13 Esawong clan, healthcare worker*

"*Bethany faiths take in sick people, with the prayer they do; they’re already curing people of their illnesses*." *E22, Efak clan, elder*

Traditional healers and healthcare workers have argued that they used to send and receive patients from different therapeutic services, particularly from western medicine to traditional healing and or the other way around. However, there does appear to be a link between different healthcare-seeking behaviours.

“*Sometimes, I used to send patients to the hospital and from the hospital used to send me patients to treat them …*” *E52 Obuk clan, traditional healer*

“*… If the patient is so seriously stricken, I must send him to the hospital …*” *E42, Ndong clan, traditional healer*

“*… not only the doctors but also the nurses …*” *E9, Anvom clan, healthcare worker*

“*… At the hospital, they say that it is not to treat here, you must go to the traditional healer …*” *E25, Nsomo clan, elder*

One of the healthcare workers made a striking comment that has a large effect on the result-seeking Western medicine in a hospital

“Witchcraft *– when you’re sick*, *if they take you to the hospital and inject you*, *you’ll die …” E9 Anvom clan healthcare worker*

“*Someone may be possessed by an evil spirit or an ancestor spirit*. *You can bring him to the western medicine many times, [but] he´ll never be recovered …*” *E17, Yesuk clan, elder*

The participant observer indicated that there is no multidisciplinary therapeutic approach to mental illness in the country. Hospitals in the healthcare system lack specialized psychiatric staff. Thus, the population is directed to other therapeutic options, such as traditional medicine or the Bethany and Pentecostal evangelical churches, to address mental disorders.

## Discussion

Fundamentally, the discussion has been formulated based on the research questions concerning the definition, signs and symptoms of Fang culture-bound syndromes. In addition, the discussion addresses an explanatory model and therapeutic attitudes about the ailments. The data were collected using a nomenclature that all participants understood. (The information provided data on subjects using common language and layout, even for healthcare workers with academic training on the biomedical, aetiological model of disease.)

The Fang folk illnesses have a major effect on social life because most involve altered behaviour or thought, and their origin is thought to lie in a magical aetiological model based on the influence of spirits, punishments, rules of conduct, and enemies. Participants formulated similar descriptions of the Fang illnesses, irrespective of geographical origin, clan, age, or group membership. Individuals from different Fang clans living in widely dispersed geographic areas (e.g., Onvang clan and Efak clan in the country in the Mongomo province and Akonibe district) and with greater economic and commercial development (e.g., the Anvom and Esanvus clans on the coast in the Kogo district) have described the Fang illnesses similarly to participants who were born in areas more exposed to Western acculturation.

Therapeutic providers (pastors, traditional healers and healthcare workers) in Equatorial Guinea and the participant observer agreed on the definition of culturally bound syndromes. Specifically, diseases that biomedicine has difficulty diagnosing and curing and/or diseases relapse/recurrence led patients to believe that they were dealing with a folk illness and to assume that there was magic involved [57–59]. The participants explained that biomedicine cannot address mental health problems because the necessary human, material, or diagnostic facilities are not available locally.

In particular, Healthcare professionals explained that some Fang culture-bound syndromes have the same signs and symptoms as diseases within a multi-causal biomedical model and that the signs and symptoms often overlap with the explanatory model. Studies in other Sub-Saharan African and South American countries such as South Africa [13], Kenya [60], and Mexico [61–63], also reported culture-bound syndromes, although the different cultures call the syndromes by different names. Van Duijl [63–64] identified a cultural syndrome known in Uganda called *okutembwa*, which is marked by being possessed by spirits and hearing voices, reminiscent of *mibili*. *Wekufü* in the Mapuche ethnic group in Chile and Argentina presents with signs of aggressiveness and acute pain [66] similar to *eluma*, a Fang culturally bound syndrome. *Wekufü* refers to an energy that can be projected from a distance and causes disease.

### Explanatory model of Fang culturally bound syndromes

It has been shown that the explanatory model of Fang culture-bound syndromes is largely a magical or religious model or a combination of both. Punishment after disregarding the law of God is the original cause of *Mikug*, *Eluma* and *Kong*. Specific concepts noted in Catholic dogma are preserved in the following three diseases: *Kong (*greedy),*Eluma* (jealousy), and *Mikug*(worshiping human bones).

In the case of *Mikug*, the participant observer linked the expression with an ancient rite that the Fang ethnic group celebrated long ago, in which the bones or skull of a person from the family, *melán*, were venerated, and family members would implore them to grant favours, to provide good crops, or to eliminate illnesses. With the arrival of the Spanish colonizers, this pagan rite was banned and became sacrilegious [67–69].

The Evuzok ethnic group from Cameroon reported a culture-bound syndrome that manifests through lethargy and disconnection from the environment similar to *kong* [70]. The current study findings indicate that the cause of *Kong* is close to the concept of greed and is linked to the wealthier or more educated social classes, professionals who work at large companies, or to people who have enriched themselves through education [70]

Breaking a traditional prohibition that elders and traditional healers left on you in a rite of passage (*dzas)*, violating a traditional rule, such as an endogamy or food taboo, or bothering the ancestors could be a cause of *Nsamadalú* [71] *or Mibili*. Van Duijl et al] [64–65] and [10] remind us that the dissociative symptoms of mental illness in Uganda are said to be due to cultural explanations, cultural obligations, uncompleted ceremonies, and attempts to resolve conflicts with spirits, rituals, ancestral spirits, witchcraft, and sociocultural conflicts such as disputes over un-repaid gifts or between landowners. In the Nande ethnic group in the Democratic Republic of the Congo, some diseases result from disobeying the rules of the ancestors [72].

Finally, Fang witchcraft likens folk illness to spiritual power, such as witches and sorcerers. In the current study, healthcare professionals and traditional healers explained how this disorder develops, attributing it to being possessed, "to suffering a type of enchantment." People are said to be manipulated by a malignant internal force, “*evu*,” that prompts the sufferer to disconnect from the environment or to exhibit antisocial behaviour. People used to use witchcraft by practising exoteric rites with the assistance of hallucinogenic substances, “which are themselves basically forces of destruction” [73–74][75–76]. Campbell [77] found that some Xhosa patients in South Africa who were diagnosed with schizophrenia attributed their mental illness to witchcraft and stated that they were possessed by an internal force, displaying a passive attitude and describing hallucinations [78][79–80].

### Healthcare-seeking behaviour of Fang culturally bound syndromes

Fang people used to visit the traditional healers when the signs and symptoms and the cultural context of illness indicated to them that witchcraft or sorcery (the participants named it “dark side”) might be affecting them or after violation of Fang traditional rules. Our findings are in line with those of other researchers; in South Africa, only a minority of patients suffering from mental illnesses were visited and diagnosed by and received treatment from Western medical providers (29%). However, most patients consulted traditional practitioners [81]. Among the Mijikenda ethnic groups on the coast of Kenya, diseases with symptoms affecting mental health, such as hallucinations and anxiety, appear to be treated only by traditional healers [60].

The above suggests that the Fang people today continue to believe in witchcraft as a causal mechanism for mental illness. In other sub-Saharan African cultures, witchcraft continues to be viewed as a causal force, although often with new symbols, new social identities and power relations][82–83] and it suggest that new ways of practising witchcraft coexist with modernity and globalized imaginaries[78–79].

In addition, participants reported that signs and symptoms of a mental illness that they have called “madness” are identified as a spiritual disorder and that it is essential to visit someone nearby to God for a cure. For Fang cosmogony, soul and body have to stand united in healthy and alive people which is true also for the Catholic faith [84]. Thus, those with mental illness must visit a Catholic clergyman for praying because prayer is the cure. Leavey et al. [84] explain that there is no doubt that spirits and demonic possession are alive and that this behaviour could be construed as a religious or spiritual disorder. In a study of mental health in Nigeria, Igbinomwanhia et al. [85]determined that more than one-half of those surveyed believed that supernatural or spiritual factors caused mental illness and that therefore their treatment should be sought from the clergy.

Equatorial Guinea is populated by many of the Catholic faith, but Pentecostal and Bethany faiths experienced rapid growth in sub-Saharan Africa in the 1990s, in part because they do not go hand-in-hand with the colonizer [86] and have become a therapeutic resource for mental illnesses. Furthermore, traditional biomedical treatments are often not available [87]. However, there is a need for treatment of antisocial behaviour because such behaviour can result in conflicts with others, financial difficulties, or relationship problems with relatives. Pentecostal faiths provide **“***traditional witchdoctors*, *medicine cults*, *charismatic prophets and solutions to the problems*” [76] and let those who are being treated preserve their African spirituality [88].

The results of the current study suggest that in culture-bound syndromes, seeking resources is affected by the belief model and magical-religious pattern of behaviour of the patients [89–92]. Traditional healing and faith practices are the preferred treatment, [93–96] but Western medicine also has an influence. The healthcare facilities often communicate, and they send patients to and receive patients from the other therapeutic facilities. The current study describes how the healthcare workers, despite the scientific knowledge and the health facilities, continue to send patients to the traditional healers because they believe that western treatment is not effective against this type of illness [97]. Other studies in geographically distant locations have discovered similar instances. In Sri Lanka, for example, using Western medicine to treat illness that does not have explanations in scientific medicine is iatrogenic [91]. Guatemalan therapists who suspect the presence of a cultural syndrome will suggest that it not be addressed by a university-trained doctor, because it is similar to watering a plant – the disease becomes “more irrigated” and the patient’s condition will worsen [98]. In the current study, the participant observer found that Equatorial Guinea has no multidisciplinary therapeutic approach to mental illness, and the hospitals in the healthcare system lack sufficient professionals who specialize in psychiatry. However, the lack of health facilities did not appear to be the cause of utilizing a traditional healer or clergy; rather the magical or symbolism origin of the disease appeared to be the cause. It became clear that the local healthcare workers understand the meaning of culture-bound syndrome, but they are convinced that Western medicine is not the right therapeutic resource. In the future, the effectiveness of Western healthcare programmes might be influenced for this reasoning.

## Limitations

The study is not without its limitations. It is difficult to describe conditions that have magical/religious aetiology, objectifying signs and symptoms of these cultural syndromes. Finally, the main researcher – the visible face of the project – was a young, white Spanish female, which could have influenced the behaviour and responses of some participants.

## Conclusions

Symbolism in African ethnicities, the supremacy of ancestors, punishment, prohibitions, rules of conduct and the principle of causality in health and disease are underlying factors in the presentation of certain diseases that in ethnopsychiatry are now referred to as “culture-bound syndromes." Elders, Fang community leaders, healthcare workers, traditional healers and pastors of evangelical faiths identified six disease processes among the Fang ethnic group in Equatorial Guinea. These processes manifest with behavioural or thought disorders and have no diagnostic label in the biomedical model; all of them share some magical or religious element as their causal agent: *eluma*, *witchcraft*, *kong*, *mibili*, *mikug*, and *nsamadalu*. In the absence of a multidisciplinary approach to mental illness in this country, the Fang ethnic group retains their cultural and religious meanings, and they use Fang traditional healing and religious rites in the churches as therapeutic resources for culture-bound diseases.

## Supporting information

S1 File Demographic characteristics English. (XLS)

S2 File Coding Summary Report E2-E58+OP. (RTF)

## Abbreviations

DSM: Diagnostic and Statistical Manual of Mental Disorders
WHO: World Health Organizations
UNDP: United Nations Development Programme
GNI: Gross National Income per capita
GDP: Gross Domestic Product
CIA: Central Intelligence Agency
INSESO: Social Health Insurance Institute
FERS: Sanitary Religious Spanish Federations
DHS: Demographic and health surveys
COREQ: Consolidated Criteria for Reporting Qualitative Research
