# Supplementary material for: Exploring the knowledge, explanatory models of illness, and patterns of healthcare-seeking behaviour of Fang culture-bound syndromes in Equatorial Guinea
Source: PLoS One. 2018 Sep 7;13(9):e0201339. doi: 10.1371/journal.pone.0201339 (PMC6128453; doi:10.1371/journal.pone.0201339)
Supplement: S2 File — (RTF) [file pone.0201339.s002.rtf]

Coding Summary Report
CULTURAL BOUND SINDROMES
Project:
Generated:
27/02/2018 13:47
Name
Initials
Coding By
IT
Inma
Total Users
 1
Elementos internos\E10
Document
References
Coverage
 2
Nodos ramificados\EXPLANATORY MODELS OF FANG CULTURAL BOUND SYNDROMES\VIOLATIONS OF TRADITIONAL RULES\DZAS
Node Coding
3,61%
Reference
Character Range
1
0,82%
Coverage
3574 - 3713
Cuando nace un niño en la tribu, había un mayor que prepara una escudilla, una escudilla con hojas platanares, bien tapadas, dan agua ahí.
Reference
Character Range
2
2,79%
Coverage
3865 - 4335
Entonces él prepara ciertos árboles, por decir, ciertas cortezas, algo que esto que llamamos DZAS, se lo prepara de acuerdo con la forma que él mismo ha visto que el niño que ha nacido; si el niño es inocente, le prepara el --------como inocente para que tenga más suerte de la forma que ha venido. Y si el niño es, digamos sabio, es decir, brujo, si es brujo, si tiene EVÚ, si el niño ha nacido con hechizo también tiene otra forma de preparar el ---------------, así.
References
Coverage
 4
Nodos ramificados\EXPLANATORY MODELS OF FANG CULTURAL BOUND SYNDROMES\VIOLATIONS OF TRADITIONAL RULES\FOOD TABOOS
Node Coding
4,00%
Reference
Character Range
1
0,94%
Coverage
554 - 713
Animal o alimento prohibido precisamente para ANVOM. No existe, pero si hay ciertos animales que comemos, que comemos con ritos, con rito; o sea, un permiso.
Reference
Character Range
2
1,35%
Coverage
746 - 974
Por ejemplo el animal civeta en Fang se llama------, usted lo sabe ¿no?, usted como Fang. Civeta, -------, lo llamamos así cualquier niño puede llegar  a la edad de cuarenta años si su padre no se le cede a comer, no puede comer.
Reference
Character Range
3
1,29%
Coverage
1184 - 1402
Coding Summary Report
Page 1 of 52


También hay otro animal ahí que se llama…jabalí, el estómago, las tripas y el estómago del jabalí, que se llama -------o cerdo, cerdo del bosque, no del pueblo. Tú no puedes comerlo si tu padre no te lo cede a comer.
Reference
Character Range
4
0,42%
Coverage
1472 - 1542
Es un mal augurio, te trae unas desgracias, enfermedades tradicionales.
References
Coverage
 2
Nodos ramificados\HEALTHCARE SEEKING BEHAVIOUR\TRADITIONAL HEALING
Node Coding
1,04%
Reference
Character Range
1
0,53%
Coverage
9718 - 9807
Cuando alguien se siente mal, de estas enfermedades tradicionales, por ejemplo, NSAMADALÚ.
Reference
Character Range
2
0,51%
Coverage
10050 - 10136
Le puede invitar al curandero, le invita a su pueblo, para que le absuelva el pueblo.
References
Coverage
 2
Nodos ramificados\SIGN AND SYMPTOM OF FANG CULTURAL BOUND SYNDROMES\WITCHCRAFT
Node Coding
4,94%
Reference
Character Range
1
1,97%
Coverage
13968 - 14300
Si en la brujería hay algún disturbio entre los asistentes, se luchan ahí, alguien se queda herido. La herida no está en su cuerpo, sino en su hechizo, la herida se queda en su hechizo, la herida se queda en el espíritu, haces cosas fuera de las normas, de repente sales corriendo, vas sin ropa por la calle, sin pelucar, sin lavar…
Reference
Character Range
2
2,97%
Coverage
14386 - 14886
Y el espíritu, si tú tienes el hechizo de salir para asistir a las brujerías, el cuerpo se queda en la cama y el espíritu es el que sale para ir a vivir otro mundo, obrar allí, bailar allí, a luchar allí. Y si desde ahí tu te quedas dañado, tu quieren encontrar tu cuerpo, el cuerpo no es más que una cáscara, entonces el espíritu es el núcleo del cuerpo humano. Entonces el cuerpo se queda inerte, no sabe nada, no sabes leer, no sabes hablar… hablas palabras sin unir…pero el espíritu está dañado.
Total References
Coverage
 10
Total Users
3,40%
 1
Coding Summary Report
Page 2 of 52


Elementos internos\E13
Document
References
Coverage
 1
Nodos ramificados\EXPLANATORY MODELS OF FANG CULTURAL BOUND SYNDROMES\PUNISHMENT
Node Coding
2,17%
Reference
Character Range
1
2,17%
Coverage
4502 - 4685
Porque los poseedores de cráneos humanos, que se dominan MIKUK, se dice de que a algún  hijo se le han cogido los obsequios y están depositados junto a esos cráneos en una enfermedad.
References
Coverage
 1
Nodos ramificados\EXPLANATORY MODELS OF FANG CULTURAL BOUND SYNDROMES\VIOLATIONS OF TRADITIONAL RULES\FOOD TABOOS
Node Coding
3,36%
Reference
Character Range
1
3,36%
Coverage
1602 - 1885
Las mujeres no se alimentaban de barro, de peces o animales que vivieran en el barro, con el peligro de tener un mal parto, un niño con deformidad, loco con gritos, llantos, con malos espíritus, una enfermedad tradicional, las mujeres no se alimentaban de… de los antílopes gigantes.
References
Coverage
 2
Nodos ramificados\HEALTHCARE SEEKING BEHAVIOUR\THE EVANGELICAL CHURCHES
Node Coding
2,54%
Reference
Character Range
1
1,59%
Coverage
6607 - 6741
Están entrando unas nuevas sectas, Iglesias, en Guinea que también dicen que curan a la gente, eso que ha oído usted hablar de esto…
Reference
Character Range
2
0,95%
Coverage
6986 - 7066
Si ellos cogen a uno enfermo, le llevan, hacen sus oraciones de este y le salva.
References
Coverage
 1
Nodos ramificados\SIGN AND SYMPTOM OF FANG CULTURAL BOUND SYNDROMES\MIKUG
Node Coding
1,65%
Reference
Character Range
1
1,65%
Coverage
4758 - 4897
Coding Summary Report
Page 3 of 52


Porque eso a veces ataca al cuerpo de la persona y le debilita, ahí en la cama sin hablar, querer comer, mirando al techo, así días y días.
Total References
Coverage
 5
Total Users
2,43%
 1
Elementos internos\E16
Document
References
Coverage
 1
Nodos ramificados\EXPLANATORY MODELS OF FANG CULTURAL BOUND SYNDROMES\VIOLATIONS OF TRADITIONAL RULES\DZAS
Node Coding
3,22%
Reference
Character Range
1
3,22%
Coverage
2759 - 3086
Preparaban una palangana con hierbas tradicionales, que ahí se metía a los niños y le daban prohibiciones, para que cuando se vaya haciendo mayorcito como cada una de estas hierbas tenía algún significado, y ahí te bañaban, es el primer agua, que te metían dentro para bañarte. En caso de cometer un error, comienza la locura.
References
Coverage
 2
Nodos ramificados\EXPLANATORY MODELS OF FANG CULTURAL BOUND SYNDROMES\VIOLATIONS OF TRADITIONAL RULES\FOOD TABOOS
Node Coding
1,33%
Reference
Character Range
1
0,75%
Coverage
883 - 959
Según dijeron nuestros abuelos, que había un animal que se llamaba NCODJEN.
Reference
Character Range
2
0,58%
Coverage
960 - 1019
Como coincide con la tribu NCODJEN.
Que no se podía comer.
References
Coverage
 2
Nodos ramificados\EXPLANATORY MODELS OF FANG CULTURAL BOUND SYNDROMES\WITCHCRAFT-SORCERY
Node Coding
2,47%
Reference
Character Range
1
2,46%
Coverage
5234 - 5483
Por ejemplo, eh…el EVU, que es lo que más domina este país, que a veces tú estás, pero el espíritu tuyo no está, ¿eh?, va a otro mundo más allá del mundo que no conocemos, y cuando viene por la mañana, ya tiene otro aspecto, sólo quiere comer carne…
Coding Summary Report
Page 4 of 52


Reference
Character Range
2
0,02%
Coverage
5529 - 5531
.
References
Coverage
 1
Nodos ramificados\HEALTHCARE SEEKING BEHAVIOUR\RELATIONSHIP BETWEEN THEM
Node Coding
0,81%
Reference
Character Range
1
0,81%
Coverage
5531 - 5613
El EVU, no se puede curar en el hospital jamás, es algo que curan los curanderos.
References
Coverage
 2
Nodos ramificados\HEALTHCARE SEEKING BEHAVIOUR\THE EVANGELICAL CHURCHES
Node Coding
4,75%
Reference
Character Range
1
2,89%
Coverage
8589 - 8882
Si tú vas una de estas sectas, tú vas a tener resultado”; ahí me llevó a una de estas sectas, yo te digo que no me dieron nada, sólo con la Fe de creer que Dios es el único que podía hacer lo imposible a posible, iba a tener una solución; no me dieron nada, sólo lo que hacían es orar por mí.
Reference
Character Range
2
1,86%
Coverage
9151 - 9340
 Y al ver este gran milagro, que en una semana yo me recuperé, solo la oración, imponer las manos, orar y tener la Fe de que esta oración que me hacen, es la que me va a ayudar, nada más.
References
Coverage
 1
Nodos ramificados\SIGN AND SYMPTOM OF FANG CULTURAL BOUND SYNDROMES\ELUMA
Node Coding
1,13%
Reference
Character Range
1
1,13%
Coverage
8274 - 8389
Era ELUMA, tenía pinchazos en la cabeza, dolores muy fuertes, gritaba a la gente, les tiraba las cosas, les mordía…
References
Coverage
 1
Nodos ramificados\SIGN AND SYMPTOM OF FANG CULTURAL BOUND SYNDROMES\MIBILI
Node Coding
2,67%
Coding Summary Report
Page 5 of 52


Reference
Character Range
1
2,67%
Coverage
4843 - 5114
Cuando siente que está sentado así, oye ruidos por los lados, cuando camina, parece ser que alguien le está siguiendo, cuando duerme ve malos sueños, ve cosas de los sepulcros, o sea, ve malos sueños; ahí considera que lo que tengo es algo tradicional, que tengo mibili.
Total References
Coverage
 10
Total Users
2,34%
 1
Elementos internos\E17
Document
References
Coverage
 1
Nodos ramificados\EXPLANATORY MODELS OF FANG CULTURAL BOUND SYNDROMES\VIOLATIONS OF TRADITIONAL RULES\FOOD TABOOS
Node Coding
0,76%
Reference
Character Range
1
0,76%
Coverage
1821 - 1961
De familia o de casa, o por nuestros temas de curas tradicionales y tal, alguien puede tener prohibido comer, a lo mejor, carne de elefante.
References
Coverage
 1
Nodos ramificados\HEALTHCARE SEEKING BEHAVIOUR\RELATIONSHIP BETWEEN THEM
Node Coding
1,76%
Reference
Character Range
1
1,76%
Coverage
11795 - 12120
Un tema que no es físico, si no espiritual, y si es espiritual no hay solución, porque puede darse el caso de una persona que está poseída por un espíritu, de un pariente o de un antepasado, lleva a ese al hospital tantas veces tú quieras, ese no se pondrá bueno. Pero llévelo a un practicante que lo arregla, te lo arregla.
References
Coverage
 1
Nodos ramificados\HEALTHCARE SEEKING BEHAVIOUR\THE EVANGELICAL CHURCHES
Node Coding
0,78%
Reference
Character Range
1
0,78%
Coverage
9282 - 9425
El hombre Fang es dualista, cree en el cuerpo y cree que sabe que hay el alma, que sabe que existen los espíritus, por eso, busca las Iglesias…
Coding Summary Report
Page 6 of 52


Total References
Coverage
 3
Total Users
1,10%
 1
Elementos internos\E18
Document
References
Coverage
 2
Nodos ramificados\EXPLANATORY MODELS OF FANG CULTURAL BOUND SYNDROMES\ACTION OF FORCES COMING FROM OTHER PEOPLE
Node Coding
1,45%
Reference
Character Range
1
1,08%
Coverage
4917 - 5033
Pero según nuestra tradición ya hay otras enfermedades que realmente se dice que alguien te ha hecho enfermar, sí.
Reference
Character Range
2
0,36%
Coverage
5283 - 5322
ELUMA, Sí, está provocada por alguien
References
Coverage
 1
Nodos ramificados\EXPLANATORY MODELS OF FANG CULTURAL BOUND SYNDROMES\VIOLATIONS OF TRADITIONAL RULES\DZAS
Node Coding
3,41%
Reference
Character Range
1
3,41%
Coverage
3120 - 3485
Cuando nace un niño hay que ver algo, esto se llama dzas, hay alguna especie de hierbas, de árboles, se buscan y se ponen dentro de una palangana y ponerle allí, primer agua que llaman la recién nacida, entonces, esto se llama dzas, es un…, es un tipo de…me refiero que protege, que protege a la persona, hasta puede llegar a ser la persona…una persona digna, sí.
References
Coverage
 2
Nodos ramificados\EXPLANATORY MODELS OF FANG CULTURAL BOUND SYNDROMES\VIOLATIONS OF TRADITIONAL RULES\FOOD TABOOS
Node Coding
2,36%
Reference
Character Range
1
1,93%
Coverage
2527 - 2733
Teníamos una serpiente que siempre nos protegía, es lo que yo sé que teníamos, una serpiente que nos protegía. Sí tú caes en el río, tú vas a encontrar algo parecido como un tipo de… un tronco de árbol...
Reference
Character Range
2
0,44%
Coverage
2950 - 2997
Coding Summary Report
Page 7 of 52


Solamente era para la protección de la tribu.
References
Coverage
 1
Nodos ramificados\HEALTHCARE SEEKING BEHAVIOUR\RELATIONSHIP BETWEEN THEM
Node Coding
1,51%
Reference
Character Range
1
1,51%
Coverage
10100 - 10262
Bueno, parece ser que hay alguna gente que uno va en hospital, va en otro sitio, va en Fang, y al final cuando va a la secta dicen que ha curado, se queda curado.
References
Coverage
 1
Nodos ramificados\HEALTHCARE SEEKING BEHAVIOUR\TRADITIONAL HEALING
Node Coding
1,22%
Reference
Character Range
1
1,22%
Coverage
5385 - 5516
¡Hombre!, hay curanderos que inmediatamente cuando se consigue que es el ELUMA, se lo cura inmediatamente, antes de un día se cura.
Total References
Coverage
 7
Total Users
1,99%
 1
Elementos internos\E19
Document
References
Coverage
 1
Nodos ramificados\EXPLANATORY MODELS OF FANG CULTURAL BOUND SYNDROMES\VIOLATIONS OF TRADITIONAL RULES\FOOD TABOOS
Node Coding
0,53%
Reference
Character Range
1
0,53%
Coverage
5725 - 5776
En mi tribu (Oserengon) no existe una prohibición…
References
Coverage
 1
Nodos ramificados\HEALTHCARE SEEKING BEHAVIOUR\TRADITIONAL HEALING
Node Coding
0,92%
Reference
Character Range
1
0,92%
Coverage
2017 - 2105
Coding Summary Report
Page 8 of 52


Entonces, que es una enfermedad tradicional, es mejor buscar al curandero para tratarle.
Total References
Coverage
 2
Total Users
0,73%
 1
Elementos internos\E2
Document
References
Coverage
 2
Nodos ramificados\EXPLANATORY MODELS OF FANG CULTURAL BOUND SYNDROMES\VIOLATIONS OF TRADITIONAL RULES\FOOD TABOOS
Node Coding
1,10%
Reference
Character Range
1
1,02%
Coverage
565 - 616
¿Hay algún animal que no puedan comer los ESAWONG.
Reference
Character Range
2
0,08%
Coverage
628 - 632
No.
References
Coverage
 2
Nodos ramificados\SIGN AND SYMPTOM OF FANG CULTURAL BOUND SYNDROMES\WITCHCRAFT
Node Coding
3,86%
Reference
Character Range
1
0,38%
Coverage
3544 - 3563
Hechicería, locura.
Reference
Character Range
2
3,48%
Coverage
3641 - 3815
Primeramente, dejan de trabajar en los trabajos, no cuidan los niños, hablan de cosas extrañas, se van por la noche, andan sin rumbo. Los ojos se ponen grandes, pero no ven.
Coding Summary Report
Page 9 of 52


Total References
Coverage
 4
Total Users
2,48%
 1
Elementos internos\E20
Document
References
Coverage
 1
Nodos ramificados\EXPLANATORY MODELS OF FANG CULTURAL BOUND SYNDROMES\ACTION OF FORCES COMING FROM OTHER PEOPLE
Node Coding
0,90%
Reference
Character Range
1
0,90%
Coverage
6955 - 7031
 ELUMA, lo que dicen que uno lanza uno así tal, esa empieza la enfermedad.
References
Coverage
 1
Nodos ramificados\EXPLANATORY MODELS OF FANG CULTURAL BOUND SYNDROMES\PUNISHMENT
Node Coding
2,29%
Reference
Character Range
1
2,29%
Coverage
6025 - 6219
Dice que después de que llegaron los colonizadores, parece que eso era lo que les atraía más, ahí estaba de dónde recibían su fuerza, entonces dijeron que se eliminara todo esto. Lo eliminaron.
References
Coverage
 1
Nodos ramificados\EXPLANATORY MODELS OF FANG CULTURAL BOUND SYNDROMES\VIOLATIONS OF TRADITIONAL RULES\FOOD TABOOS
Node Coding
3,06%
Reference
Character Range
1
3,06%
Coverage
1710 - 1970
Dice que en la preparación del melan, está prohibido comer gorila, porque decían que si lo comieran serían vencidos por los adversarios; porque siempre eran guerreros en este momentos, entonces no podían comer esto porque se considera como una animal guerrero.
References
Coverage
 1
Nodos ramificados\EXPLANATORY MODELS OF FANG CULTURAL BOUND SYNDROMES\WITCHCRAFT-SORCERY
Node Coding
1,06%
Reference
Character Range
1
1,06%
Coverage
2325 - 2415
Para ellos la enfermedad les va a tener siempre relacionado con la preparación de brujería.
Coding Summary Report
Page 10 of 52


Total References
Coverage
 4
Total Users
1,83%
 1
Elementos internos\E22
Document
References
Coverage
 1
Nodos ramificados\HEALTHCARE SEEKING BEHAVIOUR\THE EVANGELICAL CHURCHES
Node Coding
2,92%
Reference
Character Range
1
2,92%
Coverage
6817 - 7040
Actualmente suena mucho que hay muchas confesiones, Betanía, ahí va mucha gente. Betanía coge a los enfermos cantidad, con el rezo que están haciendo, a muchos suenan, muchos vienen que ya están curados de sus enfermedades.
Total References
Coverage
 1
Total Users
2,92%
 1
Elementos internos\E24
Document
References
Coverage
 3
Nodos ramificados\EXPLANATORY MODELS OF FANG CULTURAL BOUND SYNDROMES\VIOLATIONS OF TRADITIONAL RULES\DZAS
Node Coding
1,33%
Reference
Character Range
1
0,45%
Coverage
3274 - 3338
Típicamente, solamente era de que dicen: mi hijo recién nacido.
Reference
Character Range
2
0,63%
Coverage
3355 - 3445
 Un hombre que sabe arreglar los hijos, sabe preparar un hijo para que puede ser persona.
Reference
Character Range
3
0,24%
Coverage
3460 - 3494
 Persona de valor por decirlo así.
Coding Summary Report
Page 11 of 52


References
Coverage
 1
Nodos ramificados\EXPLANATORY MODELS OF FANG CULTURAL BOUND SYNDROMES\VIOLATIONS OF TRADITIONAL RULES\FOOD TABOOS
Node Coding
2,35%
Reference
Character Range
1
2,35%
Coverage
6643 - 6976
La enfermedad tradicional era estas enfermedades que… que si una mujer se fue en embarazo no hay que comer, tanto las mujeres que…el marido dice que…dio el embarazo dicen que esto no hay que comerlo, y si algún descuido, alguno de ellos come una cosa de estas, ¿eh?, entonces esto le va a perjudicar al niño o la niña, con la locura.
References
Coverage
 1
Nodos ramificados\HEALTHCARE SEEKING BEHAVIOUR\RELATIONSHIP BETWEEN THEM
Node Coding
0,58%
Reference
Character Range
1
0,58%
Coverage
6976 - 7058
Esta enfermedad pueden llevarlo al hospital pero no puede curar, hay que llevarla…
References
Coverage
 1
Nodos ramificados\HEALTHCARE SEEKING BEHAVIOUR\TRADITIONAL HEALING
Node Coding
0,62%
Reference
Character Range
1
0,62%
Coverage
7058 - 7146
Hay que llevarle al curandero Fang. Tradicionalmente se curan ahí, eso en poco tiempo.
Total References
Coverage
 6
Total Users
1,22%
 1
Elementos internos\E25
Document
References
Coverage
 1
Nodos ramificados\EXPLANATORY MODELS OF FANG CULTURAL BOUND SYNDROMES\VIOLATIONS OF TRADITIONAL RULES\DZAS
Node Coding
4,12%
Reference
Character Range
1
4,12%
Coverage
5219 - 5553
Cuando uno nace…cuando la gente nace, en la familia hay preparaciones familiares a nivel de la tradición que se hacen, y os ponen prohibiciones que a lo largo de tu vida tú no debes practicar tales cosas. Entonces, si algún miembro de la familia sale, y clandestinamente viola una de estas normas pueden aparecer enfermos mentalmente.
Coding Summary Report
Page 12 of 52


References
Coverage
 1
Nodos ramificados\EXPLANATORY MODELS OF FANG CULTURAL BOUND SYNDROMES\VIOLATIONS OF TRADITIONAL RULES\SEXUAL TABOOS
Node Coding
3,14%
Reference
Character Range
1
3,14%
Coverage
4208 - 4463
Como hemos empezado antes, el Fang conoce muchos tipos de enfermedades, por ejemplo, cuando un hermano se acuesta con su hermana o un miembro de la familia que es directo, esto se consideraba como una violación, como un pecado de las normas tradicionales.
References
Coverage
 1
Nodos ramificados\EXPLANATORY MODELS OF FANG CULTURAL BOUND SYNDROMES\WITCHCRAFT-SORCERY
Node Coding
4,12%
Reference
Character Range
1
4,12%
Coverage
1630 - 1964
Cuando vas al hospital, en el hospital te dicen que esta enfermedad no es del hospital, vete a los curanderos Fang. Y cuando llegas ahí, lo que suelen decir generalmente es: uno, que pueda que es alguien que te ha puesto la enfermedad, o que tú mismo por querer hacer manipulaciones con el arte mágico tradicional te has quedado mal.
References
Coverage
 1
Nodos ramificados\SIGN AND SYMPTOM OF FANG CULTURAL BOUND SYNDROMES\ELUMA
Node Coding
1,70%
Reference
Character Range
1
1,70%
Coverage
1478 - 1616
Hay otro tipo de enfermedades, por ejemplo, ELUMA, suelen surgir dolores costales agudos y después gritar, correr, días y días sin dormir…
References
Coverage
 2
Nodos ramificados\SIGN AND SYMPTOM OF FANG CULTURAL BOUND SYNDROMES\NSAMADALU
Node Coding
2,19%
Reference
Character Range
1
1,52%
Coverage
4464 - 4587
A causa de este hecho, de haberte acostado con tu hermana, que es lo que se llama incesto, aparecían ciertas enfermedades.
Reference
Character Range
2
0,68%
Coverage
4642 - 4697
Quedan sin voz como castigo, sin cuerpo, nsamadalu.
Coding Summary Report
Page 13 of 52


Total References
Coverage
 6
Total Users
3,05%
 1
Elementos internos\E26
Document
References
Coverage
 2
Nodos ramificados\EXPLANATORY MODELS OF FANG CULTURAL BOUND SYNDROMES\VIOLATIONS OF TRADITIONAL RULES\FOOD TABOOS
Node Coding
1,42%
Reference
Character Range
1
0,74%
Coverage
2352 - 2409
En mi familia, tenemos prohibición de no comer elefante.
Reference
Character Range
2
0,68%
Coverage
2532 - 2585
Es según nuestra tradición Fang…nos da… la riqueza.
References
Coverage
 1
Nodos ramificados\EXPLANATORY MODELS OF FANG CULTURAL BOUND SYNDROMES\WITCHCRAFT-SORCERY
Node Coding
0,72%
Reference
Character Range
1
0,72%
Coverage
3251 - 3307
Las enfermedades tradicionales son… te diré, brujería.
References
Coverage
 1
Nodos ramificados\HEALTHCARE SEEKING BEHAVIOUR\TRADITIONAL HEALING
Node Coding
0,31%
Reference
Character Range
1
0,31%
Coverage
3980 - 4004
Allí, curandería Fang.
Coding Summary Report
Page 14 of 52


Total References
Coverage
 4
Total Users
0,82%
 1
Elementos internos\E27
Document
References
Coverage
 1
Nodos ramificados\EXPLANATORY MODELS OF FANG CULTURAL BOUND SYNDROMES\VIOLATIONS OF TRADITIONAL RULES\SEXUAL TABOOS
Node Coding
0,98%
Reference
Character Range
1
0,98%
Coverage
2154 - 2245
La mujer, nunca se veía mujer por el día, por el peligro de las enfermedades de la mente.
References
Coverage
 1
Nodos ramificados\EXPLANATORY MODELS OF FANG CULTURAL BOUND SYNDROMES\WITCHCRAFT-SORCERY
Node Coding
0,64%
Reference
Character Range
1
0,64%
Coverage
4540 - 4600
Por nosotros, la enfermedad tradicional es por la brujería.
References
Coverage
 1
Nodos ramificados\HEALTHCARE SEEKING BEHAVIOUR\TRADITIONAL HEALING
Node Coding
0,59%
Reference
Character Range
1
0,59%
Coverage
4881 - 4936
¿Para curar en la familia dónde va?, te va al curandero
Total References
Coverage
 3
Total Users
0,74%
 1
Elementos internos\E28
Document
Coding Summary Report
Page 15 of 52


References
Coverage
 1
Nodos ramificados\EXPLANATORY MODELS OF FANG CULTURAL BOUND SYNDROMES\VIOLATIONS OF TRADITIONAL RULES\DZAS
Node Coding
1,99%
Reference
Character Range
1
1,99%
Coverage
1799 - 2054
Sí se pone el niño en un lugar incómodo según nuestro tiempo, madera, y luego se le pone nuestros nombres, si la persona se llega a ser un poco mayor de edad por sus faenas que se puede contraer dentro de su vida ya se trae prohibiciones de algunas cosas.
References
Coverage
 2
Nodos ramificados\EXPLANATORY MODELS OF FANG CULTURAL BOUND SYNDROMES\WITCHCRAFT-SORCERY
Node Coding
4,20%
Reference
Character Range
1
2,50%
Coverage
5311 - 5631
La enfermedad tradicional Fang es algo que… la brujería, bueno, no sé si es espiritualmente y yo no sé dónde se lo cogen, que se prepara uno cuando nace, que se le da unos tratamientos para su maniobra que lo tenemos como espíritu, no es alma, es espíritu. Ese espíritu se hace muchos movimientos que nosotros no vemos.
Reference
Character Range
2
1,70%
Coverage
6548 - 6765
Eh… esto es…ataques del espíritu, si por ejemplo yo vengo en ti y cuando el espíritu viene contra todos tus vestidos, entonces si se golpea con tus vestidos es que me quedo complicado, que lo llamamos mbo (brujería).
References
Coverage
 1
Nodos ramificados\HEALTHCARE SEEKING BEHAVIOUR\TRADITIONAL HEALING
Node Coding
1,17%
Reference
Character Range
1
1,17%
Coverage
6766 - 6916
Entonces si el espíritu estaba dañado, se traen medicamentos tradicionales en la curandería, para reparar y dejar lo que se siente y hacerlo calmar.
Total References
Coverage
 4
Total Users
2,45%
 1
Elementos internos\E3
Document
References
Coverage
 1
Nodos ramificados\EXPLANATORY MODELS OF FANG CULTURAL BOUND SYNDROMES\PUNISHMENT
Node Coding
0,61%
Coding Summary Report
Page 16 of 52


Reference
Character Range
1
0,61%
Coverage
4470 - 4629
El hombre tiene una parte inmortal que no debería morir, pero debido a la desobediencia, a las normas, entonces llegó la muerte y llegó también la enfermedad.
References
Coverage
 1
Nodos ramificados\EXPLANATORY MODELS OF FANG CULTURAL BOUND SYNDROMES\SPIRITS OR ANCESTORS
Node Coding
5,31%
Reference
Character Range
1
5,31%
Coverage
6670 - 8049
Efectivamente. Todo Fang, todo clan que puede ser una tribu o una sociedad política, tiene prohibiciones. Tiene prohibiciones como por ejemplo, hay prohibiciones que solamente pueden divulgar los iniciados, los iniciados son los que están ya introducidos en esta vida secreta, oculta; si realmente no forma parte de esta vida oculta, no puede divulgar ninguno de esos secretos. Entonces cuando alguien descubre un secreto de estos y lo divulga, eso le puede traer una consecuencia; cuando, por ejemplo, alguien ha desobedecido, ha faltado al respeto a un anciano del pueblo, alguien ha faltado al respeto a un ancestro o quizás a una reliquia, eso también le puede traer consecuencias: una enfermedad mental seria. Por lo tanto, en ese sentido el Fang entiende la enfermedad como un castigo, un castigo que puede venir de forma interna, es decir, cuando el acto es cometido por la misma persona, pero también de forma exógena cuando el castigo es hereditario, es decir, los padres hayan cometido un error con la tradición y este castigo se transmite de generación en generación, considerando lo que está escrito en la sagrada escritura: que Dios castiga desde los padres a los hijos….hasta la tercera y cuarta generación. Por eso, también el Fang entiende que cuando el padre ha cometido una falta contra la tradición, contra un espíritu, contra un ancestro, contra una reliquia.
References
Coverage
 1
Nodos ramificados\EXPLANATORY MODELS OF FANG CULTURAL BOUND SYNDROMES\VIOLATIONS OF TRADITIONAL RULES
Node Coding
0,39%
Reference
Character Range
1
0,39%
Coverage
5592 - 5692
cuando entonces el hombre desobedeció esas normas, llegó, como he dicho, la enfermedad y la muerte.
References
Coverage
 1
Nodos ramificados\EXPLANATORY MODELS OF FANG CULTURAL BOUND SYNDROMES\VIOLATIONS OF TRADITIONAL RULES\FOOD TABOOS
Node Coding
0,38%
Reference
Character Range
1
0,38%
Coverage
2046 - 2145
Bueno, efectivamente una prohibición como tal, mis progenitores no me transmitieron la información.
References
Coverage
 3
Nodos ramificados\HEALTHCARE SEEKING BEHAVIOUR\THE EVANGELICAL CHURCHES
Node Coding
5,65%
Coding Summary Report
Page 17 of 52


Reference
Character Range
1
1,14%
Coverage
5014 - 5311
También hay que entender que cuando la parte física está afectada, la parte también espiritual está afectada, por lo tanto, la razón por la cual un Fang se encuentra enfermo tiene que buscar una solución recurriendo a los curanderos, recurriendo a los adivinos, a los sacerdotes, a los religiosos.
Reference
Character Range
2
1,18%
Coverage
22037 - 22344
Nosotros entendemos que el trabajo de la Iglesia, el trabajo primordial, no consiste en la parte física, sino en la parte espiritual, y que el señor Jesucristo no vino para curar, no fue el objetivo de su nacimiento en el mundo, de su encarnación, sino es salvar a la humanidad para que…en la vida eterna.
Reference
Character Range
3
3,32%
Coverage
22617 - 23479
Pero también, considerando lo que dijo nuestro señor Jesucristo después de una curación, siempre decía: “ha curado tu Fe, te has salvado”. Lo que da a entender de que Jesucristo no llamaba a la gente: “venid, que os cure, venid, venid”, no, sino cuando alguien tenía Fe en Él y le presentaba una cierta situación como es enfermedad, inmediatamente se quedaba curada la persona. Nosotros consideramos que la curación tiene que ser algo como un milagro, como un suceso extraordinario; por lo tanto, hacemos oraciones con un hermano que se encuentre enfermo si se acerca a mí y me pide una oración por su enfermedad, no solamente por su enfermedad, por cualquier situación que puede encontrarse, que realmente él ya no puede con sus posibilidades física y mentales o económicas, inmediatamente yo puedo orar y muchas veces nosotros vivimos las respuestas divinas.
References
Coverage
 2
Nodos ramificados\HEALTHCARE SEEKING BEHAVIOUR\TRADITIONAL HEALING
Node Coding
1,82%
Reference
Character Range
1
1,29%
Coverage
8245 - 8581
Porque entiende que la medicina convencional solamente va a tener en cuenta la dolencia física, mientras tanto que esta parte oculta solo lo puede conocer la tradición. Por eso, de vez en cuando, el Fang en vez de recurrir a la medicina convencional, prefiere recurrir a la medicina tradicional que le puede resolver esa parte oculta.
Reference
Character Range
2
0,53%
Coverage
10781 - 10918
Se puede recurrir a cualquier otro medio si realmente no se ha encontrado con ningún curandero que trata el ELUMA no se puede resolver.
References
Coverage
 2
Nodos ramificados\SIGN AND SYMPTOM OF FANG CULTURAL BOUND SYNDROMES\ELUMA
Node Coding
2,19%
Reference
Character Range
1
1,66%
Coverage
10053 - 10484
Las enfermedades más frecuentes son el ELUMA por ejemplo, el ELUMA es una…según el Fang es una enfermedad proyectil, es decir, que si yo soy un enemigo tuyo y recurro a una persona poseedor del ELUMA, puede estar, por ejemplo, el puede estar a distancia de un kilómetro, te lo puede proyectar desde ahí. Entonces, y como es contacto espiritual, si tu espíritu también es apto para captar el ELUMA inmediatamente empiezas a sentir.
Coding Summary Report
Page 18 of 52


Reference
Character Range
2
0,53%
Coverage
10781 - 10918
Se puede recurrir a cualquier otro medio si realmente no se ha encontrado con ningún curandero que trata el ELUMA no se puede resolver.
References
Coverage
 2
Nodos ramificados\SIGN AND SYMPTOM OF FANG CULTURAL BOUND SYNDROMES\KONG
Node Coding
1,84%
Reference
Character Range
1
0,27%
Coverage
11431 - 11501
Bueno es... también últimamente es muy frecuente, el más conocido KONG
Reference
Character Range
2
1,57%
Coverage
11585 - 11993
Coge el espíritu de otra persona que llega hasta la muerte y cuando la persona, según ellos muere, como ya no… recupera su cuerpo y otra vez la persona tiene más…se queda en vida, pero tiene que ir a un lugar donde realmente no conoce a la persona, allí le va a hacer cualquier trabajo que él tenga necesidad, que la persona esta le realice, pero algo malo, tú apareces lento, desorientado, desconectado.
Total References
Coverage
 13
Total Users
2,27%
 1
Elementos internos\E32
Document
References
Coverage
 2
Nodos ramificados\EXPLANATORY MODELS OF FANG CULTURAL BOUND SYNDROMES\VIOLATIONS OF TRADITIONAL RULES\FOOD TABOOS
Node Coding
0,91%
Reference
Character Range
1
0,30%
Coverage
1176 - 1205
Nosotros no comemos carne ÑOK
Reference
Character Range
2
0,61%
Coverage
1237 - 1297
Es un pájaro (animal) que canta por la mañana en el bosque.
Coding Summary Report
Page 19 of 52


References
Coverage
 1
Nodos ramificados\SIGN AND SYMPTOM OF FANG CULTURAL BOUND SYNDROMES\NSAMADALU
Node Coding
1,97%
Reference
Character Range
1
1,97%
Coverage
1650 - 1843
Si te casas, los novios, a partir de un día empezarán las enfermedades, NSAMADALU, no van a las fiestas, no pueden trabajar, dolores de cabeza, no hablan, algunos se meten en la cama, sin comer.
Total References
Coverage
 3
Total Users
1,44%
 1
Elementos internos\E33
Document
References
Coverage
 2
Nodos ramificados\EXPLANATORY MODELS OF FANG CULTURAL BOUND SYNDROMES\WITCHCRAFT-SORCERY
Node Coding
2,96%
Reference
Character Range
1
0,82%
Coverage
1078 - 1127
La brujería también es causa de las enfermedades.
Reference
Character Range
2
2,13%
Coverage
2843 - 2970
La batería de la brujería es el EVU, el espíritu malo. Esa batería si tiene el poder de hacer y deshacer en cualquier momento.
References
Coverage
 1
Nodos ramificados\HEALTHCARE SEEKING BEHAVIOUR\THE EVANGELICAL CHURCHES
Node Coding
1,83%
Reference
Character Range
1
1,83%
Coverage
5215 - 5324
Los sacerdotes de la Iglesia reformada hoy en día se vuelven más curanderos que sacerdotes, curan la locura.
References
Coverage
 1
Nodos ramificados\HEALTHCARE SEEKING BEHAVIOUR\TRADITIONAL HEALING
Node Coding
2,60%
Coding Summary Report
Page 20 of 52


Reference
Character Range
1
2,60%
Coverage
2556 - 2711
El que practica brujería, o tiene una enfermedad tradicional o procedente de la brujería, tendrá que ir o le gustaría ir a la curandería tradicional, sí.
References
Coverage
 2
Nodos ramificados\SIGN AND SYMPTOM OF FANG CULTURAL BOUND SYNDROMES\WITCHCRAFT
Node Coding
2,38%
Reference
Character Range
1
1,13%
Coverage
3001 - 3068
Te transforman en un esquizofrénico, te quedas enfermo mentalmente.
Reference
Character Range
2
1,26%
Coverage
3069 - 3144
Puedes ser pobre, sin mujer, sin hijos ni dinero ni nada, no tendrá nada.
Total References
Coverage
 6
Total Users
2,44%
 1
Elementos internos\E34
Document
References
Coverage
 2
Nodos ramificados\EXPLANATORY MODELS OF FANG CULTURAL BOUND SYNDROMES\VIOLATIONS OF TRADITIONAL RULES\DZAS
Node Coding
7,53%
Reference
Character Range
1
5,13%
Coverage
4992 - 5733
Entonces había, y también ,aún existe, de que uno llama cuando nace un niño, por ejemplo, yo digo a la señora; la señora sabe buenamente que yo suelo arreglar vidas humanas como curandero, ¿no?. Pues me trae y me dice, haber tengo un niño, un bebé, de 2 semanas o menos de 2 semanas o una semana, me llama a mí y me dice “arréglame este niño para que sea persona”. Entonces no lo hacemos a solas, sino que llamamos a otros aldeanos y llevamos un palo, que llamamos “palo santo” . Entonces le llevamos allí…yo empiezo a preparar al niño porque hay dos tipos: AVALAGA, la vida de una persona enseñándole las malas vidas, ¿ah?, eso se llama AVALAGA, y lo contrario, y detrás, ¿qué haces tú? Pues dame persona para comer, para matar, es una mala.
Reference
Character Range
2
2,40%
Coverage
5734 - 6081
Predicción, esto de AVALAGA, no es bueno. Luego ACOMEYA sí porque cuando yo lo hago, lo hago en presencia de uno, dos o tres o cuatro ancianos. Y cada uno viene, pues mete su palo, otro viene mete su hierba, el otro viene mete lo suyo para que el niño, no, el bebé ¿no?, el bebé sea persona. Esta es la diferencia que hay entre ACOMEYA y AVALAGA.
Coding Summary Report
Page 21 of 52


References
Coverage
 1
Nodos ramificados\EXPLANATORY MODELS OF FANG CULTURAL BOUND SYNDROMES\VIOLATIONS OF TRADITIONAL RULES\FOOD TABOOS
Node Coding
0,18%
Reference
Character Range
1
0,18%
Coverage
926 - 952
Nosotros no comemos boa.
References
Coverage
 1
Nodos ramificados\HEALTHCARE SEEKING BEHAVIOUR\TRADITIONAL HEALING
Node Coding
0,60%
Reference
Character Range
1
0,60%
Coverage
8077 - 8163
 Primero van a las curanderías tradicionales que conocen esos tipos de enfermedades.
References
Coverage
 1
Nodos ramificados\SIGN AND SYMPTOM OF FANG CULTURAL BOUND SYNDROMES\WITCHCRAFT
Node Coding
1,15%
Reference
Character Range
1
1,15%
Coverage
7595 - 7761
Sólo la hechicería es la propia enfermedad que los Fang podemos decir que es enfermedad tradicional. No sabes dónde estás, no conoces tu familia, te llevas sin ropa.
Total References
Coverage
 5
Total Users
2,36%
 1
Elementos internos\E35
Document
References
Coverage
 3
Nodos ramificados\DEFINITION
Node Coding
3,36%
Reference
Character Range
1
1,49%
Coverage
1449 - 1527
Dile, si el Kong es una enfermedad de ahora o ya lo traían los antepasados?.
Coding Summary Report
Page 22 of 52


Reference
Character Range
2
0,53%
Coverage
1547 - 1575
Sí de ahora, antes no había.
Reference
Character Range
3
1,33%
Coverage
4934 - 5004
 Ella cree que ellos también tienen cosas tradicionales no sólo aquí.
References
Coverage
 1
Nodos ramificados\EXPLANATORY MODELS OF FANG CULTURAL BOUND SYNDROMES\ACTION OF FORCES COMING FROM OTHER PEOPLE
Node Coding
2,69%
Reference
Character Range
1
2,69%
Coverage
1661 - 1802
Lo que llamamos eluma, viene, es principio de envidia. Si alguien tiene envidia por otro y es capaz de hacerte algún mal, te puede dar eluma.
References
Coverage
 1
Nodos ramificados\EXPLANATORY MODELS OF FANG CULTURAL BOUND SYNDROMES\SPIRITS OR ANCESTORS
Node Coding
0,93%
Reference
Character Range
1
0,93%
Coverage
1326 - 1375
 Una persona poseida por malos espíritus: mibili.
Total References
Coverage
 5
Total Users
2,33%
 1
Elementos internos\E38
Document
References
Coverage
 1
Nodos ramificados\EXPLANATORY MODELS OF FANG CULTURAL BOUND SYNDROMES\PUNISHMENT
Node Coding
2,13%
Reference
Character Range
1
2,13%
Coverage
5906 - 6187
Coding Summary Report
Page 23 of 52


Ahora, como la creación va los enfermedades, las enfermedades, también van cambiando. Hay enfermedades que nosotros mismos hacemos, porque todos no somos amistades, somos enemigos de ambas partes, como kong,  que yo mismo quiero ser un rico, si tú haces medicina, tú te equivocas.
References
Coverage
 1
Nodos ramificados\HEALTHCARE SEEKING BEHAVIOUR\RELATIONSHIP BETWEEN THEM
Node Coding
0,87%
Reference
Character Range
1
0,87%
Coverage
3073 - 3187
Los curanderos lo saben; que no es enfermedad tradicional y lo hacen trasladar, ¿eh?, por eso se lleva al hospital
References
Coverage
 1
Nodos ramificados\SIGN AND SYMPTOM OF FANG CULTURAL BOUND SYNDROMES\KONG
Node Coding
0,65%
Reference
Character Range
1
0,65%
Coverage
6187 - 6272
Kong se queda medio loco, sales al bosque de noche, comes carne de lo que encuentras.
Total References
Coverage
 3
Total Users
1,21%
 1
Elementos internos\E39
Document
References
Coverage
 2
Nodos ramificados\EXPLANATORY MODELS OF FANG CULTURAL BOUND SYNDROMES\VIOLATIONS OF TRADITIONAL RULES\FOOD TABOOS
Node Coding
2,51%
Reference
Character Range
1
0,44%
Coverage
2321 - 2378
Tiene algo muy original que no puede comer: la serpiente.
Reference
Character Range
2
2,07%
Coverage
2741 - 3009
No puede comer tampoco macaco, no puede, bajo ningún concepto. Porque durante las guerras de venir cosas, según dice, tradicionalmente, que le ayudó el macaco, ¿eh?, que era un elemento que cuando se encontraban con él sabían que delante hay enemigos y volvían detrás.
Coding Summary Report
Page 24 of 52


References
Coverage
 1
Nodos ramificados\SIGN AND SYMPTOM OF FANG CULTURAL BOUND SYNDROMES\NSAMADALU
Node Coding
1,71%
Reference
Character Range
1
1,71%
Coverage
2490 - 2711
Hay una enfermedad tradicional que se llama NSAMADALU, le ocurre a la novia o al novio, quedan aislados de la gente, débiles, sin hablar, llorando, a veces gritan a los mayores y los niños con problemas de salud serios.
Total References
Coverage
 3
Total Users
2,11%
 1
Elementos internos\E4
Document
References
Coverage
 1
Nodos ramificados\DEFINITION
Node Coding
0,32%
Reference
Character Range
1
0,32%
Coverage
3474 - 3559
Son enfermedades que no pueden ser tratadas, curadas o diagnosticadas por la ciencia.
References
Coverage
 1
Nodos ramificados\EXPLANATORY MODELS OF FANG CULTURAL BOUND SYNDROMES\ACTION OF FORCES COMING FROM OTHER PEOPLE
Node Coding
0,34%
Reference
Character Range
1
0,34%
Coverage
3295 - 3385
Entonces la enfermedades tradicionales provocadas por la gente, es decir, con la brujería.
References
Coverage
 1
Nodos ramificados\EXPLANATORY MODELS OF FANG CULTURAL BOUND SYNDROMES\SPIRITS OR ANCESTORS
Node Coding
0,29%
Reference
Character Range
1
0,29%
Coverage
7671 - 7748
Entonces lo que puede causar que el espíritu tenga dolencia es otro espíritu.
Coding Summary Report
Page 25 of 52


References
Coverage
 1
Nodos ramificados\EXPLANATORY MODELS OF FANG CULTURAL BOUND SYNDROMES\VIOLATIONS OF TRADITIONAL RULES\FOOD TABOOS
Node Coding
2,06%
Reference
Character Range
1
2,06%
Coverage
1270 - 1821
Sí, en realidad era una prohibición, o sea, de los abuelos, de los antepasados, pero en principio no lo respeto. Era un… es un pajarito de color algo así…que no se cuál es el nombre del pájaro … ñok…que decían que no se come porque canta cuando escucha ruidos, ese pájaro multiplica por cada año, o sea, tiende a construir nido, tiende a vivir junto a la gente, entonces prohibieron a que no se lo comiesen, representaba como una forma de… de, o sea, de cercanía del pájaro al hombre y no se tenía que comer, era algo como guardián para la tribu esa.
References
Coverage
 4
Nodos ramificados\HEALTHCARE SEEKING BEHAVIOUR\THE EVANGELICAL CHURCHES
Node Coding
2,99%
Reference
Character Range
1
0,55%
Coverage
12725 - 12872
Y como Dios dice que “ lo que es imposible para el hombre, es posible para Dios”, en ese momento Él también te puede sanar lejos de ir al hospital.
Reference
Character Range
2
0,86%
Coverage
22421 - 22650
Pero un caso que ni la medicina puede intervenir, o sea, ya ha hecho lo que puede y ya no se puede hacer nada, ahí se puede presentar a Dios que intervenga, y muchos casos, yo he visto, que el Señor los ha resuelto, muchos casos.
Reference
Character Range
3
0,33%
Coverage
23242 - 23331
Los casos más usuales que nosotros tratamos ahora en Guinea, son los casos espiritistas.
Reference
Character Range
4
1,26%
Coverage
24426 - 24762
Porque obtenía dentro malos espíritus, mibili, malos pensamientos, solamente para nosotros lo que hacemos es pedir al Señor que si hay un mal espíritu que se manifieste y que se huya de ese cuerpo, y vemos como la persona, o sea, se manifiesta y se van los espíritus, entonces son los casos más corrientes que hay, que la Iglesia trata.
References
Coverage
 1
Nodos ramificados\HEALTHCARE SEEKING BEHAVIOUR\TRADITIONAL HEALING
Node Coding
0,33%
Reference
Character Range
1
0,33%
Coverage
3384 - 3473
Coding Summary Report
Page 26 of 52


 Para nosotros los Fang, se trataba de manera tradicional que es mediante los curanderos.
Total References
Coverage
 9
Total Users
1,05%
 1
Elementos internos\E40
Document
References
Coverage
 1
Nodos ramificados\EXPLANATORY MODELS OF FANG CULTURAL BOUND SYNDROMES\PUNISHMENT
Node Coding
0,99%
Reference
Character Range
1
0,99%
Coverage
5664 - 5752
Si no que la gente lo van o adquiriendo o trayendo por ambición al dinero, a la riqueza…
References
Coverage
 1
Nodos ramificados\EXPLANATORY MODELS OF FANG CULTURAL BOUND SYNDROMES\VIOLATIONS OF TRADITIONAL RULES\DZAS
Node Coding
5,02%
Reference
Character Range
1
5,02%
Coverage
1315 - 1763
Cuando un niño nace, le pueden decir que no coma una cierta cosa, que no haga unas ciertas cosas…y cuando va creciendo creciendo, durante un tiempo deja de hacer lo que le han dicho que no haga. Eso de alguna que otra forma le tendrá que manifestar como una enfermedad, es lo que se llama enfermedad tradicional, porque o se lo han hecho por malicia, o porque esa misma persona no ha sabido mantener las prohibiciones que le han dado, cuando nació.
References
Coverage
 2
Nodos ramificados\HEALTHCARE SEEKING BEHAVIOUR\TRADITIONAL HEALING
Node Coding
1,81%
Reference
Character Range
1
1,26%
Coverage
3930 - 4042
La enfermedad que alguien haya hecho algo que no debe hacerse, se llevaba directamente al curandero tradicional.
Reference
Character Range
2
0,55%
Coverage
5866 - 5915
Sino a través de la curandería se puede detectar.
Coding Summary Report
Page 27 of 52


References
Coverage
 1
Nodos ramificados\SIGN AND SYMPTOM OF FANG CULTURAL BOUND SYNDROMES\KONG
Node Coding
0,98%
Reference
Character Range
1
0,98%
Coverage
5577 - 5664
Que no se sabe cómo, porque viene de la parte de Camerún, no es algo típico de Guinea.
Total References
Coverage
 5
Total Users
2,20%
 1
Elementos internos\E41
Document
References
Coverage
 1
Nodos ramificados\EXPLANATORY MODELS OF FANG CULTURAL BOUND SYNDROMES\SPIRITS OR ANCESTORS
Node Coding
2,51%
Reference
Character Range
1
2,51%
Coverage
1538 - 1662
Mira, la enfermedad tradicional puede ser por muchas causas, por ejemplo, somos espiritistas, puede decir que tal espíritu.
References
Coverage
 1
Nodos ramificados\HEALTHCARE SEEKING BEHAVIOUR\RELATIONSHIP BETWEEN THEM
Node Coding
5,10%
Reference
Character Range
1
5,10%
Coverage
3786 - 4038
Últimamente una enfermera de esas…Inés, una mujer cayó en el piso y ella sabía que yo podía…le rompió toda la cadera, ya tenía hemorragia, y ya en el hospital es cuando…una de esas que me conocen, le dicen vete ahí donde salen ,trajeron esa mujer aquí.
Total References
Coverage
 2
Total Users
3,80%
 1
Elementos internos\E42
Document
Coding Summary Report
Page 28 of 52


References
Coverage
 2
Nodos ramificados\EXPLANATORY MODELS OF FANG CULTURAL BOUND SYNDROMES\PUNISHMENT
Node Coding
2,93%
Reference
Character Range
1
1,46%
Coverage
3083 - 3212
El hombre quiere tener mucha riqueza, cuando ha equivocado lo que le dijeron, ya no se puede ser rico porque ya está en error.
Reference
Character Range
2
1,46%
Coverage
3223 - 3352
Nuestra riqueza pasaba a nuestro niños, tener muchos hijos, tener muchas mujeres, no es tener dinero o poder para matar a otros.
References
Coverage
 2
Nodos ramificados\EXPLANATORY MODELS OF FANG CULTURAL BOUND SYNDROMES\VIOLATIONS OF TRADITIONAL RULES\DZAS
Node Coding
4,08%
Reference
Character Range
1
2,39%
Coverage
2256 - 2467
La enfermedad tradicional de un Fang, ¿eh?, significa si uno tiene uno prohibido, que sus padres y sus abuelos le han dicho en comer esto, es tratamiento, ¿eh?, se llama dzas. Al principio cuando el niño nace…
Reference
Character Range
2
1,69%
Coverage
2549 - 2698
Cuando le están bañando, lo han puesto todo medicamento, le dice  o su madre o su padre cuando estas cosas quedan prohibidas no comer, o no acercar.
References
Coverage
 4
Nodos ramificados\EXPLANATORY MODELS OF FANG CULTURAL BOUND SYNDROMES\VIOLATIONS OF TRADITIONAL RULES\FOOD TABOOS
Node Coding
1,44%
Reference
Character Range
1
0,60%
Coverage
1345 - 1398
Y hay alguna concreta de su tribu que no pueda comer.
Reference
Character Range
2
0,08%
Coverage
1530 - 1537
Coding Summary Report
Page 29 of 52


Culebra
Reference
Character Range
3
0,25%
Coverage
1664 - 1686
Tortuga yo no comer.
Reference
Character Range
4
0,51%
Coverage
2993 - 3038
Si el hombre come lo que le tiene prohibido.
References
Coverage
 1
Nodos ramificados\HEALTHCARE SEEKING BEHAVIOUR\RELATIONSHIP BETWEEN THEM
Node Coding
0,39%
Reference
Character Range
1
0,39%
Coverage
8646 - 8680
Si está grave lo pasa al hospital.
Total References
Coverage
 9
Total Users
2,21%
 1
Elementos internos\E43
Document
References
Coverage
 1
Nodos ramificados\DEFINITION
Node Coding
1,45%
Reference
Character Range
1
1,45%
Coverage
15582 - 15819
En todas las razas, en todos los continentes, en todo…en todo el mundo hay enfermedades tradicionales, pero lo que difiere es en la manera de concebirlo y la cultura, la cultura que ya tiene cierto país, cierto…cierta tribu, cierta raza.
References
Coverage
 1
Nodos ramificados\EXPLANATORY MODELS OF FANG CULTURAL BOUND SYNDROMES\ACTION OF FORCES COMING FROM OTHER PEOPLE
Node Coding
0,28%
Coding Summary Report
Page 30 of 52


Reference
Character Range
1
0,28%
Coverage
1146 - 1192
Que puede ser un ser igual que te quiere mal.
References
Coverage
 1
Nodos ramificados\EXPLANATORY MODELS OF FANG CULTURAL BOUND SYNDROMES\PUNISHMENT
Node Coding
0,53%
Reference
Character Range
1
0,53%
Coverage
1058 - 1145
Considera la enfermedad como un mal impuesto por alguien que puede ser un ser superior.
Total References
Coverage
 3
Total Users
0,76%
 1
Elementos internos\E44
Document
References
Coverage
 1
Nodos ramificados\EXPLANATORY MODELS OF FANG CULTURAL BOUND SYNDROMES\VIOLATIONS OF TRADITIONAL RULES\FOOD TABOOS
Node Coding
0,98%
Reference
Character Range
1
0,98%
Coverage
1222 - 1401
Alguna persona que ha equivocado alguna norma tradicional, es la persona que se enferma; por eso prohibían muchas…muchos animales: no se debe comer tal, no se debe hacer tal cosa…
References
Coverage
 2
Nodos ramificados\HEALTHCARE SEEKING BEHAVIOUR\RELATIONSHIP BETWEEN THEM
Node Coding
1,93%
Reference
Character Range
1
0,64%
Coverage
10144 - 10261
Pero lo único que habría que explicar sería es que hay muy pocos centros sanitarios cualificados, ese es el problema.
Reference
Character Range
2
1,29%
Coverage
17507 - 17742
Coding Summary Report
Page 31 of 52


Porque la caída…la cooperación, la salida de la cooperación, la pérdida de funcionamiento de los centros de salud y todos los puestos de salud en todas las áreas naturales, ha hecho de que aumente más la idea de curanderos tradicional.
References
Coverage
 2
Nodos ramificados\HEALTHCARE SEEKING BEHAVIOUR\THE EVANGELICAL CHURCHES
Node Coding
0,92%
Reference
Character Range
1
0,76%
Coverage
12637 - 12775
Eso está entrando mucho en nuestro medio, yo creo que sí porque hay sacerdotes que están tratando a la gente con mucha matiz tradicional.
Reference
Character Range
2
0,16%
Coverage
12863 - 12893
Creo que tiene mucha adoración.
References
Coverage
 2
Nodos ramificados\HEALTHCARE SEEKING BEHAVIOUR\TRADITIONAL HEALING
Node Coding
0,99%
Reference
Character Range
1
0,54%
Coverage
3842 - 3940
La gran concepción que tiene la gente del pueblo es que debe reunir a un curandero tradicional, y…
Reference
Character Range
2
0,45%
Coverage
7233 - 7315
Las causas no naturales, hacen que muchas veces se inclina a la cura tradicional.
References
Coverage
 2
Nodos ramificados\SIGN AND SYMPTOM OF FANG CULTURAL BOUND SYNDROMES\GENERAL
Node Coding
1,12%
Reference
Character Range
1
0,42%
Coverage
6497 - 6573
Los síntomas… serían los mismo que una enfermedad científicamente detectado.
Coding Summary Report
Page 32 of 52


Reference
Character Range
2
0,70%
Coverage
6843 - 6970
Una persona que tiene alucinaciones, porque para nosotros, tradicionalmente, el sueño malo significa una enfermedad tradicional.
Total References
Coverage
 9
Total Users
1,19%
 1
Elementos internos\E45
Document
References
Coverage
 1
Nodos ramificados\EXPLANATORY MODELS OF FANG CULTURAL BOUND SYNDROMES\ACTION OF FORCES COMING FROM OTHER PEOPLE
Node Coding
0,53%
Reference
Character Range
1
0,53%
Coverage
952 - 1043
Porque para los Fang, creen de que una persona puede poner, puede dejar al otro enfermar.
References
Coverage
 2
Nodos ramificados\EXPLANATORY MODELS OF FANG CULTURAL BOUND SYNDROMES\PUNISHMENT
Node Coding
0,76%
Reference
Character Range
1
0,39%
Coverage
843 - 910
Es decir, que la persona haya hecho un mal o tiene muchos pecados.
Reference
Character Range
2
0,37%
Coverage
11197 - 11261
(Kong) en nuestro medio se atribuye más gente un poco adinerada.
References
Coverage
 1
Nodos ramificados\EXPLANATORY MODELS OF FANG CULTURAL BOUND SYNDROMES\SPIRITS OR ANCESTORS
Node Coding
0,38%
Reference
Character Range
1
0,38%
Coverage
10060 - 10126
Coding Summary Report
Page 33 of 52


Le dicen que tiene mibili, un ancestro le ha entrado en el cuerpo.
References
Coverage
 1
Nodos ramificados\HEALTHCARE SEEKING BEHAVIOUR\RELATIONSHIP BETWEEN THEM
Node Coding
1,17%
Reference
Character Range
1
1,17%
Coverage
14069 - 14271
Lo primero que se tiene que hace es asegurar los recursos humanos, es decir, formar a las persona porque en el sistema no existe suficiente personal capacitado para llevar lo que es un sistema de salud.
References
Coverage
 1
Nodos ramificados\SIGN AND SYMPTOM OF FANG CULTURAL BOUND SYNDROMES\MIBILI
Node Coding
1,36%
Reference
Character Range
1
1,36%
Coverage
10127 - 10361
Esa persona modifica la voz, empieza a hablar de otra forma, imitando, o dice que un abuelo que murió en la familia está en su cuerpo y esa persona sí va a hablar en ese minuto. Entonces, puede ser un hombre y habla voz de una chica.
Total References
Coverage
 6
Total Users
0,84%
 1
Elementos internos\E46
Document
References
Coverage
 3
Nodos ramificados\EXPLANATORY MODELS OF FANG CULTURAL BOUND SYNDROMES\PUNISHMENT
Node Coding
1,89%
Reference
Character Range
1
0,46%
Coverage
2261 - 2337
El motivo de las enfermedades tradicionales: el pecado de la desobediencia.
Reference
Character Range
2
0,57%
Coverage
8983 - 9078
Se usan los cráneos humanos y esos huesos que están usando ahí, nos trae muchas enfermedades.
Coding Summary Report
Page 34 of 52


Reference
Character Range
3
0,86%
Coverage
15596 - 15738
Sí. Otras religiones curan, pero con el secreto de estar sin pecar. Tal como le he dicho, que el pecado… la enfermedad es origen del pecado.
References
Coverage
 1
Nodos ramificados\EXPLANATORY MODELS OF FANG CULTURAL BOUND SYNDROMES\VIOLATIONS OF TRADITIONAL RULES\FOOD TABOOS
Node Coding
0,12%
Reference
Character Range
1
0,12%
Coverage
1660 - 1680
No comen la tortuga.
References
Coverage
 4
Nodos ramificados\EXPLANATORY MODELS OF FANG CULTURAL BOUND SYNDROMES\VIOLATIONS OF TRADITIONAL RULES\SEXUAL TABOOS
Node Coding
2,22%
Reference
Character Range
1
0,70%
Coverage
1320 - 1436
Sólo lo que tienen como prohibición es que ningún Oserengon puede casarse con una de Oserengon porque es su hermana.
Reference
Character Range
2
0,53%
Coverage
6857 - 6945
El hombre y la mujer no tienen que juntarse o hacer fornicación cuando ya es nuevo día.
Reference
Character Range
3
0,56%
Coverage
7218 - 7310
Hay personas que se juntas con las mujeres en tiempo de menstruación, y esto es muy peligro.
Reference
Character Range
4
0,43%
Coverage
13153 - 13224
Fornicando con una pariente, eso también trae enfermedad…corre peligro.
Coding Summary Report
Page 35 of 52


Total References
Coverage
 8
Total Users
1,41%
 1
Elementos internos\E47
Document
References
Coverage
 1
Nodos ramificados\DEFINITION
Node Coding
0,32%
Reference
Character Range
1
0,32%
Coverage
4276 - 4359
Pero en realidad se aplica el tratamiento para curar a esa persona, que no se cura.
References
Coverage
 1
Nodos ramificados\EXPLANATORY MODELS OF FANG CULTURAL BOUND SYNDROMES\ACTION OF FORCES COMING FROM OTHER PEOPLE
Node Coding
0,31%
Reference
Character Range
1
0,31%
Coverage
1312 - 1394
Eluma son acciones mágicas dirigidas a dañar a través de fuerzas sobrenaturales.
References
Coverage
 1
Nodos ramificados\EXPLANATORY MODELS OF FANG CULTURAL BOUND SYNDROMES\PUNISHMENT
Node Coding
0,41%
Reference
Character Range
1
0,41%
Coverage
764 - 870
Para ellos la génesis o el origen de la enfermedad procede de las fuerzas sobrenaturales, castigo de Dios.
References
Coverage
 5
Nodos ramificados\EXPLANATORY MODELS OF FANG CULTURAL BOUND SYNDROMES\WITCHCRAFT-SORCERY
Node Coding
4,80%
Reference
Character Range
1
0,28%
Coverage
691 - 763
Lo atribuyen a la brujería, y no lo creen mucho en la propia naturaleza.
Coding Summary Report
Page 36 of 52


Reference
Character Range
2
0,30%
Coverage
11105 - 11183
EVU es el hechizo que…que… que le meten a uno en el cuerpo, es como un “chif”.
Reference
Character Range
3
0,08%
Coverage
11425 - 11446
Como una segunda alma.
Reference
Character Range
4
0,89%
Coverage
11470 - 11703
Cuando la persona hechizada cuando duerme, sale, sale fuera. El cuerpo está, pero sale su alma, sale para… más allá. Yo no sé si es el mundo celeste, no sé qué, para hacer la brujería. Pero el cuerpo está ahí, pero sale su espíritu.
Reference
Character Range
5
3,25%
Coverage
12040 - 12887
Bueno, la parte mala, AkIAK significa compromiso, ¿me entiende, no?. Bueno, yo le preparo para que usted tenga muchos  hijos, le preparo para que usted tenga mucho dinero o usted tenga mucho poder a cambio de que usted me ofrezca una cosa. Eso es lo que se llama AYAK, eso es lo que se llama AVALAGA-. Eso es una parte, MALA de la brujería.
Y hay lo que se llama AKOMEYA  que significa… Viene de la palabra AKOME significa piedra. Quiere decir, prepararle a una persona con ayuda de hierbas, ciertas fuerzas sobrenaturales, para que sea grande; es la planta buena. Sea para los Fang, para que uno sea grande tiene que tener AKOMEYA y los que están para comer, a sus hermanos tal, tienen la parte AVALAGA, O sea la buena es AKOMEYA y la mala AKIAK, AVALAGA que es para comer carne humana y que lo ponen en situaciones a cambio de ofrecer otra vida.
References
Coverage
 2
Nodos ramificados\HEALTHCARE SEEKING BEHAVIOUR\RELATIONSHIP BETWEEN THEM
Node Coding
1,98%
Reference
Character Range
1
0,56%
Coverage
2096 - 2241
Se curan solamente a través de la medicina tradicional, porque hay enfermedades tradicionales que con la medicina científica no podemos salvar.
Reference
Character Range
2
1,43%
Coverage
5330 - 5702
Nuestro pueblo es campesino, más del 70% de la población es campesino, es campesina. Y los campesinos…la cobertura sanitaria no llega allí, ese es el primer obstáculo. Al no llegar allí la cobertura sanitaria, la asistencia médico- farmacéutica, lo primero que hacen es recibir medicina tradicional o a la medicina mágica- religiosa, casi absorbe la mayoría de los casos.
References
Coverage
 1
Nodos ramificados\HEALTHCARE SEEKING BEHAVIOUR\THE EVANGELICAL CHURCHES
Node Coding
0,32%
Coding Summary Report
Page 37 of 52


Reference
Character Range
1
0,32%
Coverage
22831 - 22914
Porque dicen ellos para que uno se cure hay que creer, primero, debe creer en Dios.
References
Coverage
 1
Nodos ramificados\SIGN AND SYMPTOM OF FANG CULTURAL BOUND SYNDROMES\GENERAL
Node Coding
0,29%
Reference
Character Range
1
0,29%
Coverage
4201 - 4276
Los síntomas pueden ser parecidos a una determinada enfermedad científica.
Total References
Coverage
 12
Total Users
1,20%
 1
Elementos internos\E48
Document
References
Coverage
 1
Nodos ramificados\DEFINITION
Node Coding
1,32%
Reference
Character Range
1
1,32%
Coverage
8354 - 8520
Las enfermedades tradicionales también están en otros países, que ha ido a Camerún en dos sitios diferentes durante cuatro meses y ha visto las mismas enfermedades.
References
Coverage
 2
Nodos ramificados\EXPLANATORY MODELS OF FANG CULTURAL BOUND SYNDROMES\ACTION OF FORCES COMING FROM OTHER PEOPLE
Node Coding
0,93%
Reference
Character Range
1
0,25%
Coverage
11746 - 11778
Existen enfermedades como ELUMA.
Reference
Character Range
2
0,68%
Coverage
11803 - 11888
Coding Summary Report
Page 38 of 52


 No me gusta lo que hace Esperanza o tiene algo que yo añoro, le lanzo la enfermedad.
References
Coverage
 1
Nodos ramificados\EXPLANATORY MODELS OF FANG CULTURAL BOUND SYNDROMES\VIOLATIONS OF TRADITIONAL RULES\DZAS
Node Coding
3,91%
Reference
Character Range
1
3,91%
Coverage
6471 - 6963
Existen enfermedades tradicionales, de país y ha dado el ejemplo de cuando  alguien nace, ya sea su padre, ya sea su madre, la familia del padre o de la madre
te pueden coger, a base de preparados, dzas…preguntan que quieres hacer  qué quieres ser en tu vida y te terminan de preparar con productos naturales, después te dan algunas prohibiciones que durante tu vida, no hay que hacer esto, no hay que hacer no sé qué, uno puede saltar alguna de esas prohibiciones, si lo haces caes enfermo.
References
Coverage
 1
Nodos ramificados\SIGN AND SYMPTOM OF FANG CULTURAL BOUND SYNDROMES\GENERAL
Node Coding
0,72%
Reference
Character Range
1
0,72%
Coverage
7128 - 7218
Dice que más o menos son los mismos síntomas que las enfermedades de la medicina moderna.
Total References
Coverage
 5
Total Users
1,72%
 1
Elementos internos\E49
Document
References
Coverage
 1
Nodos ramificados\DEFINITION
Node Coding
3,10%
Reference
Character Range
1
3,10%
Coverage
8517 - 8812
Las enfermedades tradicionales existen en otros países. Los medicamentos que vienen AHORA en forma de comprimidos, llevan nuestras medicinas, esa gente viene aquí a comprar esas cortezas porque esos árboles no existen allí y preparar sus medicinas , luego tendrán allí las mismas enfermedades.
References
Coverage
 1
Nodos ramificados\EXPLANATORY MODELS OF FANG CULTURAL BOUND SYNDROMES\SPIRITS OR ANCESTORS
Node Coding
1,07%
Reference
Character Range
1
1,07%
Coverage
8149 - 8251
Coding Summary Report
Page 39 of 52


Los antepasados pueden comunicarse con los vivos y si estás mal con ellos te pueden traer enfermedad.
References
Coverage
 1
Nodos ramificados\EXPLANATORY MODELS OF FANG CULTURAL BOUND SYNDROMES\VIOLATIONS OF TRADITIONAL RULES\FOOD TABOOS
Node Coding
1,03%
Reference
Character Range
1
1,03%
Coverage
5601 - 5699
cuando un hermano y una hermana mantenían relaciones sexuales, o dos personas de la misma familia…
References
Coverage
 1
Nodos ramificados\HEALTHCARE SEEKING BEHAVIOUR\RELATIONSHIP BETWEEN THEM
Node Coding
1,20%
Reference
Character Range
1
1,20%
Coverage
7659 - 7773
La brujería causa enfermedad, nvo no se cura en el hospital, los médicos del hospital no pueden curar la brujería.
References
Coverage
 2
Nodos ramificados\HEALTHCARE SEEKING BEHAVIOUR\TRADITIONAL HEALING
Node Coding
1,52%
Reference
Character Range
1
0,74%
Coverage
4622 - 4692
Te envía a un curandero, si tienes esta enfermedad que te envían ELUMA.
Reference
Character Range
2
0,78%
Coverage
7774 - 7848
Cuando alguien tiene una enfermedad de la brujería va dónde el curandero.
Total References
Coverage
 6
Total Users
1,59%
 1
Elementos internos\E50
Document
Coding Summary Report
Page 40 of 52


References
Coverage
 1
Nodos ramificados\EXPLANATORY MODELS OF FANG CULTURAL BOUND SYNDROMES\ACTION OF FORCES COMING FROM OTHER PEOPLE
Node Coding
1,75%
Reference
Character Range
1
1,75%
Coverage
1040 - 1242
Aquí tenemos una enfermedad, eso viene dentro del cuerpo pero no es un hueso del esqueleto humano, que sobra, pero esto está puesto por unos magiosos, lo preparan pero muy sencillo, para preparar eso.
References
Coverage
 1
Nodos ramificados\EXPLANATORY MODELS OF FANG CULTURAL BOUND SYNDROMES\PUNISHMENT
Node Coding
0,37%
Reference
Character Range
1
0,37%
Coverage
3316 - 3359
Por otra parte por la envidia de los demás.
References
Coverage
 1
Nodos ramificados\EXPLANATORY MODELS OF FANG CULTURAL BOUND SYNDROMES\SPIRITS OR ANCESTORS
Node Coding
0,35%
Reference
Character Range
1
0,35%
Coverage
3803 - 3843
Otros dicen que son espíritus que vienen.
References
Coverage
 2
Nodos ramificados\EXPLANATORY MODELS OF FANG CULTURAL BOUND SYNDROMES\WITCHCRAFT-SORCERY
Node Coding
5,87%
Reference
Character Range
1
4,36%
Coverage
5888 - 6390
Si le ha pegado fuertemente en el espíritu, una a la otra en una lucha en la brujería, no puede faltar que 2 ó 3 días después una de las 2 tiene que caer enferma.
No eran sueños, ni tampoco ocurre en la realidad estaban en la brujería, la brujería es un magia fang, a veces uno va contra otro, el primero tiene que dañarse al otro, si los 2 se quedan igual, entonces uno muere y el otro también puede morir, si uno ha sido pero el más famoso, mata al otro, o sea que son luchas, cosas de espíritus.
Reference
Character Range
2
1,51%
Coverage
7383 - 7557
Una persona dañada así en el espíritu, evú es un espíritu, puede ser maligno, entonces que hace manifiesta que he hecho esta cosa he ido a la brujería, y me he quedado dañado.
Coding Summary Report
Page 41 of 52


Total References
Coverage
 5
Total Users
2,09%
 1
Elementos internos\E51
Document
References
Coverage
 1
Nodos ramificados\DEFINITION
Node Coding
2,86%
Reference
Character Range
1
2,86%
Coverage
5319 - 5490
Las enfermedades tradicionales fang como la brujería, eluma, mikug… otras bueno se llamarán de otras formas, también pueden estar en otros países Camerún, Francia, España.
Total References
Coverage
 1
Total Users
2,86%
 1
Elementos internos\E52
Document
References
Coverage
 2
Nodos ramificados\EXPLANATORY MODELS OF FANG CULTURAL BOUND SYNDROMES\ACTION OF FORCES COMING FROM OTHER PEOPLE
Node Coding
3,47%
Reference
Character Range
1
1,50%
Coverage
1094 - 1165
Las enfermedades tradicionales son producto, que son obra de la gente.
Reference
Character Range
2
1,97%
Coverage
1225 - 1318
Un grupo de gente se han sentado, se han preparado  una enfermedad y la han echado a alguien.
References
Coverage
 1
Nodos ramificados\EXPLANATORY MODELS OF FANG CULTURAL BOUND SYNDROMES\SPIRITS OR ANCESTORS
Node Coding
1,12%
Reference
Character Range
1
1,12%
Coverage
1165 - 1218
Coding Summary Report
Page 42 of 52


Del enfrentamiento entre los antepasados y el enfermo.
References
Coverage
 1
Nodos ramificados\HEALTHCARE SEEKING BEHAVIOUR\RELATIONSHIP BETWEEN THEM
Node Coding
2,05%
Reference
Character Range
1
2,05%
Coverage
4626 - 4723
En algunos casos envía pacientes al hospital y desde el hospital le envían pacientes para curar.
References
Coverage
 1
Nodos ramificados\SIGN AND SYMPTOM OF FANG CULTURAL BOUND SYNDROMES\KONG
Node Coding
3,09%
Reference
Character Range
1
3,09%
Coverage
2907 - 3053
Kong es una enfermedad nueva, aunque sea tradicional, bueno no es puramente tradicional, es importada de la magia de otros países, como Camerún.
Total References
Coverage
 5
Total Users
2,43%
 1
Elementos internos\E53
Document
References
Coverage
 1
Nodos ramificados\HEALTHCARE SEEKING BEHAVIOUR\THE EVANGELICAL CHURCHES
Node Coding
2,78%
Reference
Character Range
1
2,78%
Coverage
2960 - 3073
SI HA ESCUCHADO QUE LAS IGLESIAS CURAN LAS ENFERMEDADES La Iglesia reformada rezan con la gente que está enferma.
References
Coverage
 1
Nodos ramificados\HEALTHCARE SEEKING BEHAVIOUR\TRADITIONAL HEALING
Node Coding
5,14%
Reference
Character Range
1
5,14%
Coverage
3439 - 3648
Coding Summary Report
Page 43 of 52


La brujería se puede curar. Generalmente se curan como ellos saben lo que les pasa, después de hablar el curandero les hace cura tradicional y también hacen sacrificios con animales  patos, gallinas, cerdos…
Total References
Coverage
 2
Total Users
3,96%
 1
Elementos internos\E55
Document
References
Coverage
 1
Nodos ramificados\EXPLANATORY MODELS OF FANG CULTURAL BOUND SYNDROMES\VIOLATIONS OF TRADITIONAL RULES\FOOD TABOOS
Node Coding
1,66%
Reference
Character Range
1
1,66%
Coverage
2996 - 3056
Las mujeres no pueden comer un animal como perro, como lobo.
References
Coverage
 1
Nodos ramificados\SIGN AND SYMPTOM OF FANG CULTURAL BOUND SYNDROMES\MIKUG
Node Coding
5,82%
Reference
Character Range
1
5,82%
Coverage
3395 - 3605
Una enfermedad tradicional era que si una mujer veía los ídolos, los cráneos se ponía muy enferma, el cuerpo paralizado, sin mover las piernas, los brazos incluso llegaba a morir si no decía que lo había visto.
Total References
Coverage
 2
Total Users
3,74%
 1
Elementos internos\E56
Document
References
Coverage
 1
Nodos ramificados\EXPLANATORY MODELS OF FANG CULTURAL BOUND SYNDROMES\PUNISHMENT
Node Coding
3,30%
Reference
Character Range
1
3,30%
Coverage
3623 - 3748
Coding Summary Report
Page 44 of 52


Anteriormente la enfermedad tradicional era nseng, lo que ahora llamáis pecado y si cometías un pecado tu te quedas enfermo.
References
Coverage
 2
Nodos ramificados\EXPLANATORY MODELS OF FANG CULTURAL BOUND SYNDROMES\SPIRITS OR ANCESTORS
Node Coding
2,27%
Reference
Character Range
1
0,79%
Coverage
2552 - 2582
Vienen por los malos espíritus.
Reference
Character Range
2
1,48%
Coverage
2634 - 2690
Sabe usted que hay espíritus buenos y espíritus malos.
References
Coverage
 1
Nodos ramificados\EXPLANATORY MODELS OF FANG CULTURAL BOUND SYNDROMES\VIOLATIONS OF TRADITIONAL RULES\DZAS
Node Coding
7,00%
Reference
Character Range
1
7,00%
Coverage
3058 - 3323
Anteriormente se metía al niño en una palangana con hierbas esto para dotarle de las oportunidades que pueda haber, para que tenga facilidades para hacer las cosas en sus tiempos de vida. Esto se llamaba “nchas”, algunos que se lo han dejado lo siguen practicando.
References
Coverage
 1
Nodos ramificados\EXPLANATORY MODELS OF FANG CULTURAL BOUND SYNDROMES\VIOLATIONS OF TRADITIONAL RULES\FOOD TABOOS
Node Coding
3,46%
Reference
Character Range
1
3,46%
Coverage
371 - 502
Los YENKEN no comemos gorila, porque tenían una señal, según la forma que guerraban porque nuestros abuelos guerraban sin sentido.
Total References
Coverage
 5
Total Users
4,01%
 1
Elementos internos\E57
Document
Coding Summary Report
Page 45 of 52


References
Coverage
 1
Nodos ramificados\EXPLANATORY MODELS OF FANG CULTURAL BOUND SYNDROMES\VIOLATIONS OF TRADITIONAL RULES\FOOD TABOOS
Node Coding
5,13%
Reference
Character Range
1
5,13%
Coverage
1182 - 1414
Nuestra tribu no come gorilas,
Después de que hablen los gorilas, de que gritan en el bosque, en esos días no falta que se muera uno de la tribu Ngama. Esto es como un aviso. Los gorilas gritan cuando alguien está próximo a morir.
References
Coverage
 1
Nodos ramificados\HEALTHCARE SEEKING BEHAVIOUR\TRADITIONAL HEALING
Node Coding
1,26%
Reference
Character Range
1
1,26%
Coverage
2730 - 2787
 Los curanderos fang curan lo que se llama la hechicería.
Total References
Coverage
 2
Total Users
3,20%
 1
Elementos internos\E58
Document
References
Coverage
 1
Nodos ramificados\EXPLANATORY MODELS OF FANG CULTURAL BOUND SYNDROMES\VIOLATIONS OF TRADITIONAL RULES\DZAS
Node Coding
10,88%
Reference
Character Range
1
10,88%
Coverage
1150 - 1505
Cuando me nací yo si, según me contaba mi papá, iban al bosque cogían hierbas, cogían árboles y lo meten en una palangana o en un cubo, echa agua, lo ponen al sol y el niño recién nacido lo meten ahí, y durante un mes bañándole. Lo llaman “nchas”, aquí te marcan las prohibiciones que no debes acercar en vida, y si lo haces, corres peligro de la locura.
References
Coverage
 1
Nodos ramificados\EXPLANATORY MODELS OF FANG CULTURAL BOUND SYNDROMES\VIOLATIONS OF TRADITIONAL RULES\FOOD TABOOS
Node Coding
1,62%
Reference
Character Range
1
1,62%
Coverage
803 - 856
había una culebra que se llama ANVON,  no lo comían,
Coding Summary Report
Page 46 of 52


References
Coverage
 1
Nodos ramificados\EXPLANATORY MODELS OF FANG CULTURAL BOUND SYNDROMES\WITCHCRAFT-SORCERY
Node Coding
1,84%
Reference
Character Range
1
1,84%
Coverage
1854 - 1914
Vienen por la hechicería, algunos se curan y pueden morir.
Total References
Coverage
 3
Total Users
4,78%
 1
Elementos internos\E7
Document
References
Coverage
 1
Nodos ramificados\EXPLANATORY MODELS OF FANG CULTURAL BOUND SYNDROMES\ACTION OF FORCES COMING FROM OTHER PEOPLE
Node Coding
1,36%
Reference
Character Range
1
1,36%
Coverage
3921 - 4050
¿El Kong?, ah… mis hermanos que andan de Gabón a Camerún, y los que están en las empresas grandes, lo han visto con los blancos.
Total References
Coverage
 1
Total Users
1,36%
 1
Elementos internos\E8
Document
References
Coverage
 1
Nodos ramificados\EXPLANATORY MODELS OF FANG CULTURAL BOUND SYNDROMES\PUNISHMENT
Node Coding
3,41%
Reference
Character Range
1
3,41%
Coverage
4723 - 5094
La enfermedad tradicional es algo como… si mi padre me dice que no hay que comer chimpancé, esto se prohíbe dentro de nuestra familia, bueno si yo no escucho esa palabra, me voy al otro pueblo, matan ahí un chimpancé y empiezo a comer, lo que va a ocurrir ahí yo mismo tendré la culpa porque me dijeron: no hay que hacer eso, yo voy a padecer una enfermedad tradicional.
Coding Summary Report
Page 47 of 52


References
Coverage
 2
Nodos ramificados\EXPLANATORY MODELS OF FANG CULTURAL BOUND SYNDROMES\VIOLATIONS OF TRADITIONAL RULES\FOOD TABOOS
Node Coding
2,61%
Reference
Character Range
1
0,55%
Coverage
2585 - 2645
Para nosotros, en mi propia familia, no comemos chimpancé.
Reference
Character Range
2
2,06%
Coverage
2714 - 2938
Era una señal tradicional de los guerreros Fang. Cuando van a hacer guerra en otra tribu y ese chimpancé viene, levanta en medio del camino, le da una señal, ellos tienen que volver atrás, si van adelante todos van a morir.
References
Coverage
 1
Nodos ramificados\HEALTHCARE SEEKING BEHAVIOUR\THE EVANGELICAL CHURCHES
Node Coding
1,43%
Reference
Character Range
1
1,43%
Coverage
10606 - 10762
La Iglesia Betanía y está orando con persona que están débiles tanto en espíritu como en la carne. Pero hay muchas personas que salen ahí con mucha energía.
Total References
Coverage
 4
Total Users
2,48%
 1
Elementos internos\E9
Document
References
Coverage
 2
Nodos ramificados\DEFINITION
Node Coding
2,38%
Reference
Character Range
1
1,45%
Coverage
1060 - 1257
La enfermedad para un fang es cuando uno tiene una dolencia física o espiritual porque de forma espiritual, las enfermedades tradicionales no duelen pero hay una imaginación de que está enfermo.
Reference
Character Range
2
0,93%
Coverage
6126 - 6252
Coding Summary Report
Page 48 of 52


QUE EL KONG ES UNA ENFERMEDAD IMPORTADA DE OTROS PAÍSES, COMO NUEVA, ANTES NO SE HABLABA DE KONG COMO ENFERMEDAD TRADICIONAL
References
Coverage
 3
Nodos ramificados\EXPLANATORY MODELS OF FANG CULTURAL BOUND SYNDROMES\ACTION OF FORCES COMING FROM OTHER PEOPLE
Node Coding
3,96%
Reference
Character Range
1
0,42%
Coverage
1483 - 1540
Un familiar que le quiere mal y le ha dado una enfermedad.
Reference
Character Range
2
1,24%
Coverage
1541 - 1709
Y cuando va al curandero le dice que tal familiar le ha dado la enfermedad, suele ser muchas veces un familiar muy cercano, o un enemigo que le ha dado esta enfermedad.
Reference
Character Range
3
2,31%
Coverage
7605 - 7919
Dicen que algunos antepasados pueden causar enfermedad por ejemplo la brujería, si alguien que ha dado el hechizo a un niño y este niño, no le ha pagado de lo que le pedía,  y esta persona ha muerto, entonces dicen que va a seguirle hasta que le pague, entonces el niño no podrá vivir tranquilo hasta que lo diga…
References
Coverage
 1
Nodos ramificados\EXPLANATORY MODELS OF FANG CULTURAL BOUND SYNDROMES\SPIRITS OR ANCESTORS
Node Coding
0,56%
Reference
Character Range
1
0,56%
Coverage
1257 - 1333
Son espíritus malignos que a veces molestan, entonces lo llamamos MibiLi.
References
Coverage
 2
Nodos ramificados\HEALTHCARE SEEKING BEHAVIOUR\RELATIONSHIP BETWEEN THEM
Node Coding
2,14%
Reference
Character Range
1
0,88%
Coverage
5806 - 5926
La hechicería, Evu, la brujería cuando estás enfermo de esta enfermedad si te llevan al hospital y lo inyectan se muere.
Coding Summary Report
Page 49 of 52


Reference
Character Range
2
1,26%
Coverage
9398 - 9569
Ellos mismos aconsejan a los pacientes que esta enfermedad no es enfermedad del hospital, tienes que ir a los curanderos, y no solamente los médicos, hasta las enfermeras.
References
Coverage
 1
Nodos ramificados\HEALTHCARE SEEKING BEHAVIOUR\THE EVANGELICAL CHURCHES
Node Coding
0,75%
Reference
Character Range
1
0,75%
Coverage
10923 - 11025
Las personas van mucho a estas iglesias para que les hagan oraciones, curas de los espíritus malignos.
References
Coverage
 1
Nodos ramificados\HEALTHCARE SEEKING BEHAVIOUR\TRADITIONAL HEALING
Node Coding
0,43%
Reference
Character Range
1
0,43%
Coverage
5927 - 5986
Otra enfermedad como el KONG, tampoco lo llevan al hospital.
Total References
Coverage
 10
Total Users
1,70%
 1
Elementos internos\OBSERVACION PARTICIPANTE
Document
References
Coverage
 1
Nodos ramificados\DEFINITION
Node Coding
6,98%
Reference
Character Range
1
6,98%
Coverage
118 - 764
Toda persona que presenta un conjunto de signos y síntomas sin etiqueta diagnóstica por la medicina occidental, bien, porque no existe una respuesta favorable al tratamiento de la medicina occidental, bien, porque no se dispone de los recursos humanos, materiales o diagnósticos necesarios para buscar una alternativa terapéutica de confianza, en estos casos, se trama una etiología con origen en la sospecha, en la culpa, en el odio, en las rencillas entre familias, en el disconfort con los antepasados y el paciente muere, después de multitudinarias y originales terapias de la medicina mágica-religiosa y se etiqueta como fang folk illness.
References
Coverage
 3
Nodos ramificados\EXPLANATORY MODELS OF FANG CULTURAL BOUND SYNDROMES\PUNISHMENT
Node Coding
9,20%
Coding Summary Report
Page 50 of 52


Reference
Character Range
1
1,71%
Coverage
2941 - 3099
No obstante, está muy arraigada las creencia entre la población fang, de que la enfermedad tiene su origen en las fuerzas sobrenaturales: “Dios nos castiga”.
Reference
Character Range
2
3,62%
Coverage
3110 - 3445
Existía un rito tradicional, conocido como melan, en el que se veneraba el cráneo o los restos óseos de un antepasado bondadoso de la familia. Cuentan los mayores, que una vez consumado su fallecimiento se decapitaba y se guardaba el cráneo para pedirle favores, salud para los enfermos, buenas cosechas, lluvia en temporada de sequía.
Reference
Character Range
3
3,88%
Coverage
4737 - 5096
Algunas de las cajitas de madera, genialmente decoradas y talladas, que custodiaban las reliquias humanas “osarios”, llamadas en fang “bieri”, están expuestas en las vitrinas en la actualidad en el Museo Nacional de Antropología en Madrid.  Los misioneros españoles las retiraron de las manos de los nativos, por considerarlo un rito pagano y un sacrilegio.
References
Coverage
 1
Nodos ramificados\EXPLANATORY MODELS OF FANG CULTURAL BOUND SYNDROMES\VIOLATIONS OF TRADITIONAL RULES\SEXUAL TABOOS
Node Coding
5,93%
Reference
Character Range
1
5,93%
Coverage
5123 - 5672
Nsamadalu, el incesto, está marcado, como enfermedad tradicional, en la población fang, no sólo entre hermanos y hermanas consanguíneos, sino también entre miembros de la misma tribu, cuando una pareja fang, se conoce, se preguntan por el nombre de la tribu de cada uno, si el resultado fuera que pertenecen a la misma tribu, aunque han nacido en lugares distintos, la relación debe cesar, y nunca progresa. Todas las tribus fang coinciden en no mantener relaciones sexuales con mujeres u hombres que pertenecen a la misma tribu, a la misma familia.
References
Coverage
 1
Nodos ramificados\HEALTHCARE SEEKING BEHAVIOUR\THE EVANGELICAL CHURCHES
Node Coding
6,43%
Reference
Character Range
1
6,43%
Coverage
7601 - 8196
En cuanto a los cultos religiosos, como parte del itinerario terapéutico de los fang en Guinea, desde la observación participante, se ha podido constatar, la existencia de numerosas Iglesias Evangélicas repartidas por toda la geografía nacional, con diferentes nombres registrados: Iglesia Nazareno, Resurrección, del Séptimo día, Betania. Conectan con la población a partir de una situación de enfermedad,  a veces le proporcionan ayuda material o personal, trabajan con la empatía, escucha, a veces consuelo moral…, hasta que ganan su confianza y captan su voluntad, consiguiendo un adepto.
References
Coverage
 2
Nodos ramificados\HEALTHCARE SEEKING BEHAVIOUR\TRADITIONAL HEALING
Node Coding
6,37%
Reference
Character Range
1
2,38%
Coverage
5885 - 6105
Coding Summary Report
Page 51 of 52


Existen enfermedades mentales que, deberían ser abordadas por psiquiatras o psicólogos, y que caen en un vacío terapéutico, al carecer de suficientes unidades de psiquiatría o de psiquiatras, en los hospitales de Guinea.
Reference
Character Range
2
4,00%
Coverage
6786 - 7156
Se da la circunstancia de que los hospitales públicos carecen, sobre todo de profesionales sanitarios especialistas en enfermedades mentales de primer nivel, que resuelvan los primeros estadíos de la enfermedad o sus complicaciones, por lo que se observan numerosas altas voluntarias, interrumpiendo el tratamiento que habían comenzado y se trasladan a las curanderías.
References
Coverage
 1
Nodos ramificados\SIGN AND SYMPTOM OF FANG CULTURAL BOUND SYNDROMES\KONG
Node Coding
7,02%
Reference
Character Range
1
7,02%
Coverage
1280 - 1930
Desde la observación participante, se ha comprobado que, cuando un paciente padece signos o síntomas como dolor de cabeza, alucinaciones, alteraciones del estado de ánimo, de la movilidad o el lenguaje, se catalogaban en la medicina tradicional como KONG, después de una visita a la medicina occidental, se comprobaba que el paciente sufría enfermedades como la depresión, SIDA en estadíos muy avanzados de la enfermedad, que cursan con ceguera, encefalitis, meningitis, demencias, sífilis en su último estadío. Siempre más afectada la clase social de mayor status económico, con una vida social y de ocio más activa, con mayor número de contactos.
References
Coverage
 1
Nodos ramificados\SIGN AND SYMPTOM OF FANG CULTURAL BOUND SYNDROMES\WITCHCRAFT
Node Coding
9,45%
Reference
Character Range
1
9,45%
Coverage
2033 - 2908
Personas diagnosticadas de brujería, hechicería…, como enfermedad tradicional, por los curanderos o personas cercanas al entorno del paciente, comentaban que, habían estado en contacto con prácticas nocturnas, que pertenecían a sectas con ritos de canibalismo. Se les observaba desconectados con el medio, algunos conservaban cierta midriasis/miosis pupilar, sequedad de boca, verbalizaban alucinaciones visuales, su aspecto aparecía desaliñado. Se intuía que, estarían bajo los efectos de ciertas sustancias alucinógenas, probablemente, extraídas de cortezas de los árboles o de plantas, mezcladas con venenos, con fluidos de animales salvajes, existen zonas geográficas bien conocedoras de este tipo de sustancias en Guinea Ecuatorial, sin olvidar la presión, el estrés que tendrían que sufrir al verse practicar rituales socialmente prohibidos o religiosamente castigados.
Total References
Coverage
 10
Total Users
7,34%
 1
Coding Summary Report
Page 52 of 52
